# IKKα promotes lung adenocarcinoma growth through ERK signaling activation via DARPP-32-mediated inhibition of PP1 activity

**DOI:** 10.1038/s41698-023-00370-3

**Published:** 2023-03-25

**Authors:** Sk. Kayum Alam, Li Wang, Zhu Zhu, Luke H. Hoeppner

**Affiliations:** 1 The Hormel Institute, University of Minnesota, Austin, MN USA; 2 Masonic Cancer Center, University of Minnesota, Minneapolis, MN USA

**Keywords:** Non-small-cell lung cancer, Phosphorylation

## Abstract

Non-small cell lung cancer (NSCLC) accounts for 80–85% cases of lung cancer cases. Diagnosis at advanced stages is common, after which therapy-refractory disease progression frequently occurs. Therefore, a better understanding of the molecular mechanisms that control NSCLC progression is necessary to develop new therapies. Overexpression of IκB kinase α (IKKα) in NSCLC correlates with poor patient survival. IKKα is an NF-κB-activating kinase that is important in cell survival and differentiation, but its regulation of oncogenic signaling is not well understood. We recently demonstrated that IKKα promotes NSCLC cell migration by physically interacting with dopamine- and cyclic AMP-regulated phosphoprotein, Mr 32000 (DARPP-32), and its truncated splice variant, t-DARPP. Here, we show that IKKα phosphorylates DARPP-32 at threonine 34, resulting in DARPP-32-mediated inhibition of protein phosphatase 1 (PP1), subsequent inhibition of PP1-mediated dephosphorylation of ERK, and activation of ERK signaling to promote lung oncogenesis. Correspondingly, IKKα ablation in human lung adenocarcinoma cells reduced their anchorage-independent growth in soft agar. Mice challenged with IKKα-ablated HCC827 cells exhibited less lung tumor growth than mice orthotopically administered control HCC827 cells. Our findings suggest that IKKα drives NSCLC growth through the activation of ERK signaling via DARPP-32-mediated inhibition of PP1 activity.

## Introduction

Lung cancer is the second most frequently diagnosed cancer in both men and women and the leading cause of cancer-related deaths worldwide, with an estimated 2.2 million new cases and 1.8 million deaths per year^1,2^. Non-small cell lung cancer (NSCLC) is the most common type of lung cancer and accounts for 85% of total diagnoses^3^. Substantial improvements in the application of predictive biomarkers, smoking cessation, and modification of current treatment paradigms have led to notable progress in managing NSCLC and have transformed outcomes for many patients^4–6^. However, the 5-year relative survival of lung cancer patients is dismal (22.9%) due to the emergence of therapy-resistant disease and metastasis^7,8^. Therefore, improving the general understanding of disease biology, implementing screening programs to diagnose patients early, and identifying alternative treatment strategies to circumvent treatment-refractory disease progression is required to improve the lung cancer survival rate. Here, we introduce a new mechanism for the molecular regulation of oncogenic signaling that builds upon current knowledge of lung cancer biology and may inform the development of novel anticancer therapies.

IκB (inhibitor of nuclear factor kappa B) kinase α (IKKα), a serine/threonine protein kinase, is encoded by the conserved helix-loop-helix ubiquitous kinase (*CHUK*) gene^9^. Phosphorylation of IκBα, a nuclear factor-κB (NF-κB) inhibitor, by IKKα and IKKβ, catalytical subunits of the IKK complex, promotes IκBα protein degradation, which initiates nuclear translocation of NF-κB dimers. In the nucleus, NF-κB functions as a transcription factor to regulate immunity, infection, lymphoid organ/cell development, cell death/growth, and tumorigenesis^9–13^. In noncanonical signaling, NF-κB–inducing kinase activates IKKα protein via phosphorylation upon activation of upstream membrane-bound receptors by their cognate ligands. Active IKKα then phosphorylates and cleaves the p100 protein to generate p52, which complexes with the RelB NF-κB subunit, resulting in nuclear translocation of the p52/RelB dimer to regulate several immune functions, including lymphoid organ development, the priming function of dendritic cells, B-cell survival, generation, and maintenance of effector- and memory- T cells, and antiviral innate immunity^9,14,15^.

The tumor-promoting role of IKKα has been documented in breast, prostate, nonmelanoma skin, and lung cancer^16–18^. Aberrant overexpression of IKKα protein is associated with decreased patient survival and promotes the growth of lung adenocarcinoma; it may therefore be used as a biomarker to predict clinical response in lung adenocarcinoma patients^19^. In a separate study, investigators showed that overexpression of cytosolic and nuclear IKKα protein promotes NSCLC cell proliferation, survival, and migration by activating the ERK, p38/MAPK, and mammalian target of rapamycin (mTOR) cell signaling pathways. Additionally, activation of protumorigenic cell signaling pathways depends on the subcellular localization of IKKα^18^. Although the role of IKKα in promoting cancer has been well established in the context of lung cancer driven by *Kras*-activating mutations, it may have tumor-suppressing activity: in a *Kras*^*G12D*^-driven spontaneous mouse model of NSCLC, lung-specific *Ikkα* deletion induced by intratracheally injected adenovirus-Cre recombinase promoted NSCLC initiation and growth by elevating the expression of inflammatory cytokines and chemokines, including NF-κB targets^20^. We sought to understand the role of IKKα protein overexpression in tumor growth and progression in *Kras*-wild-type NSCLC.

Dopamine- and cyclic AMP-regulated phosphoprotein, Mr 32000 (DARPP-32), is primarily expressed in the brain, including the caudate nucleus, cerebral cortex, and striatum. It acts as a downstream signaling molecule through dopamine receptor 1 (D_1_R) and is negatively regulated by dopamine receptor 2 (D_2_R) and glutamate signaling^21–23^. Phosphorylation of DARPP-32 in response to cAMP in dopamine-responsive nerve tissue attenuates protein phosphatase 1 (PP1) activity, affecting the regulation of several cell signaling pathways^24^. Although expression of DARPP-32 proteins is typically restricted to neuronal cell types in the brain, DARPP-32 and its truncated isoform t-DARPP are aberrantly overexpressed in many types of cancer, including lung cancer^25–31^. t-DARPP, which was originally discovered in gastric cancer tissues, lacks the N-terminal domain responsible for modulating PP1 function^28^. It is phosphorylated by cyclin-dependent kinase (CDK) 1 and 5 and activates protein kinase A (PKA), thereby conferring resistance to trastuzumab, a HER2-targeted anticancer agent, via sustained signaling through the phosphatidylinositol-4,5-bisphosphate 3-kinase (PI3K)/AKT pathway^32,33^. Since this discovery, the DARPP-32 and t-DARPP isoforms overexpressed in breast, colon, esophageal, gastric, pancreas, prostate, lung, and ovarian cancer tissues have been shown to activate robust anti-apoptotic signaling through the activation of the AKT and ERK cell signaling pathways; to increase metabolism by forming a complex with the insulin-like growth factor 1 receptor (IGF1R); and to promote cell survival in the presence of receptor tyrosine kinase inhibitors, including gefitinib and trastuzumab^25–27,29,30,34–37^. Our previous work, which serves as the rationale for this current study, revealed that DARPP-32 isoforms increase NSCLC cell migration via increasing the expression of NF-κB2–controlled migratory genes by establishing a direct physical interaction with IKKα^25^. However, the precise role of the DARPP-32/IKKα complex in regulating NSCLC progression has yet to be determined.

In this study, we report that the IKKα protein inhibits PP1 function through phosphorylation of the full-length DARPP-32 protein at the Thr-34 position. Pharmacologic inhibition of PP1 activates ERK cell signaling pathways, leading to NSCLC growth promotion in vitro. Furthermore, we show in an orthotopic mouse model that depletion of IKKα protein reduces NSCLC growth. Taken together, our findings suggest that IKKα protein directly phosphorylates full-length DARPP-32 protein to stimulate oncogenic kinase activity through the inhibition of PP1 function to promote NSCLC growth and oncogenesis.

## Results

### Phosphorylation of DARPP-32 at Thr-34 is regulated by IKKα

**Fig. 1:**
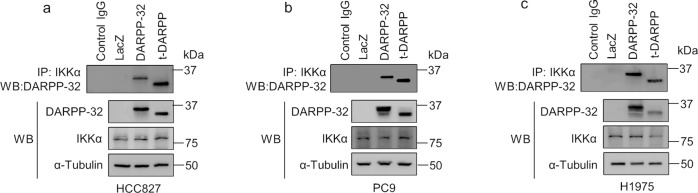
IKKα physically associates with DARPP-32 isoforms. **a-c** Human lung adenocarcinoma cell lines HCC827 (**a**), PC9 (**b**), and H1975 (**c**) stably overexpressing FLAG-tagged human DARPP-32 isoforms were lysed and subjected to immunoprecipitation using anti-IKKα antibodies. Immunoprecipitated lysates were separated in SDS-PAGE and immunoblotted with antibodies against IKKα, FLAG (that detects exogenously overexpressed DARPP-32), and α-tubulin (loading control).

**Fig. 2:**
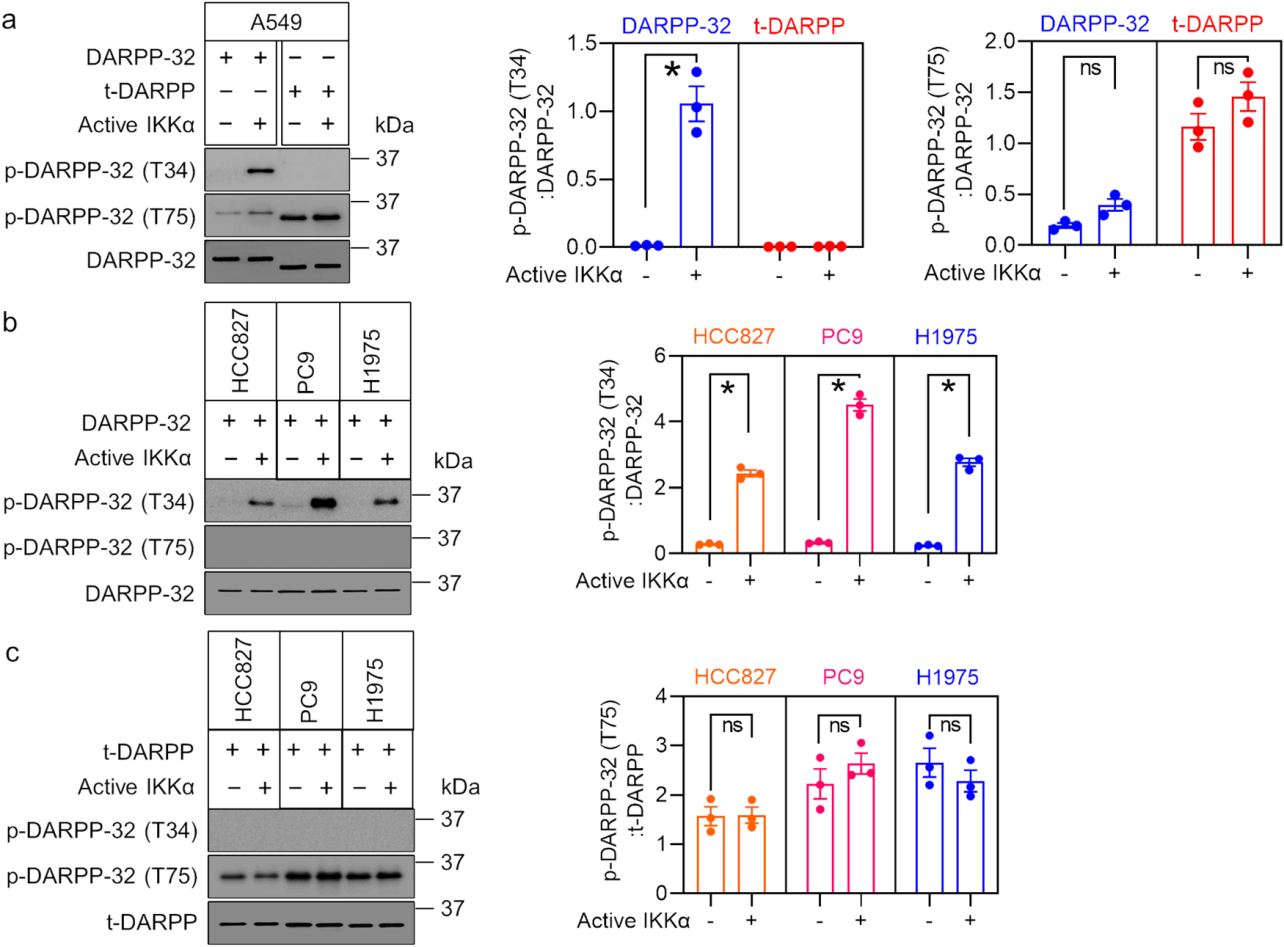
IKKα phosphorylates DARPP-32 at Thr-34. **a** Human A549 cell lines stably overexpressing FLAG-tagged human DARPP-32 isoforms (DARPP-32 and t-DARPP) were lysed and subjected to immunoprecipitation using anti-FLAG antibody–conjugated agarose beads. Immunoprecipitated lysates were used to perform nonradioactive in vitro kinase assays following incubation with commercially available active IKKα protein. At the end, the reaction mixtures were subjected to immunoblotting using antibodies against DARPP-32 phosphorylated on Thr-34 or Thr-75 and total DARPP-32 protein. **b**, **c** Human HCC827, PC9, and H1975 lung adenocarcinoma cell lines retrovirally transduced with either FLAG-tagged human DARPP-32 (**b**) or t-DARPP (**c**) cDNA plasmids were lysed, immunoprecipitated, incubated with active IKKα protein, and subjected to western blotting using anti-phospho (Thr-34 or Thr-75) DARPP-32 and anti-DARPP-32 antibodies. Data from one experimental replicate are shown. The experiments were repeated three times independently; each circle in a bar represents one experiment. Error bars indicate SEM. *P < 0.05; ns not significant.

Given our prior observation that the physical association between IKKα and DARPP-32 promotes NSCLC cell migration^25^, we postulated that DARPP-32 can be phosphorylated by the kinase function of IKKα. To test our hypothesis, we first performed immunoprecipitation experiments in three human NSCLC cell lines, HCC827, PC9, and H1975, which confirmed that IKKα establishes a direct physical interaction with DARPP-32 (Fig. 1a–c). We next performed nonradioactive in vitro kinase assays using commercially available kinase-active IKKα protein. Briefly, DARPP-32 and its short isoform, t-DARPP, were purified from lysates of four different human lung adenocarcinoma cell lines, A549, HCC827, PC9, and H1975, using anti-FLAG M2 affinity beads and then incubated with purified IKKα protein in kinase assay buffers containing ATP. Reaction end products were subjected to immunoblotting with anti-phospho DARPP-32 (both T34 and T75) and -total DARPP-32 antibodies. Our western blotting results confirm that purified full-length DARPP-32 protein (but not t-DARPP) serves directly as a substrate for IKKα (Fig. 2a, b). Based on our results, it is evident that IKKα phosphorylates full-length DARPP-32 protein at the Thr-34 position (Fig. 2a, b). As expected, IKKα does not phosphorylate t-DARPP because the Thr-34 residue is absent in t-DARPP protein since t-DARPP lacks the first 36 amino acids present in full-length DARPP-32 protein (Fig. 2a, c). However, the presence of strong signals on the immunoblot using anti-phospho DARPP-32 (T75) suggests that t-DARPP is phosphorylated at Thr-75 by an unknown endogenous kinase(s) (Fig. 2a, c). In summary, our results indicate that IKKα physically associates with DARPP-32 protein and phosphorylates full-length DARPP-32 protein at the Thr-34 position. While our findings suggest that full-length DARPP-32 protein is not phosphorylated at Thr-75 by IKKα, we were unable to test whether IKKα phosphorylates DARPP-32 at positions other than Thr-34 due to the lack of availability of anti-phospho DARPP-32 antibodies specific for other sites.

### Increased expression of p-ERK is regulated by IKKα via DARPP-32/PP1α signaling

**Fig. 3:**
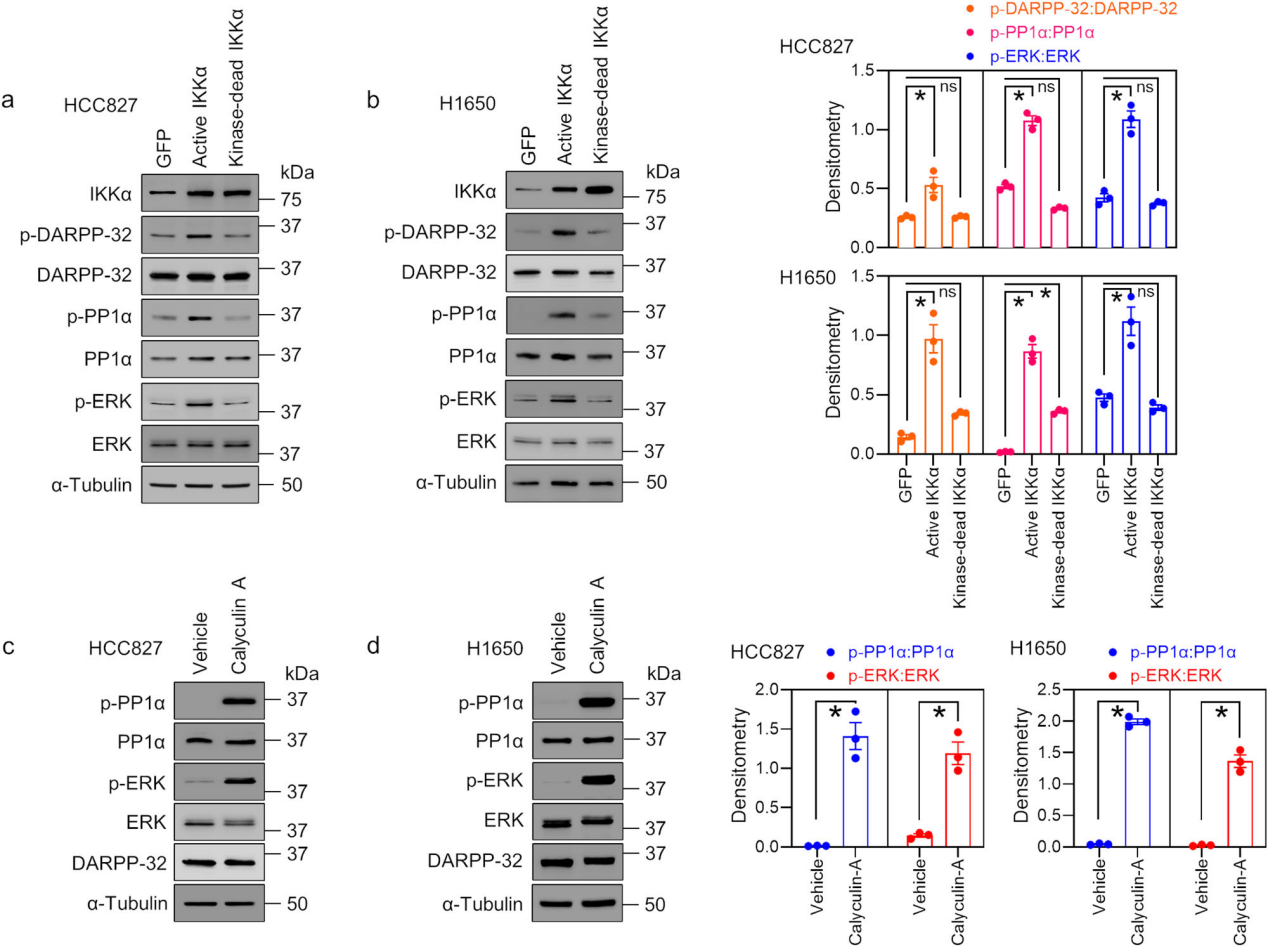
Overexpression of constitutively active IKKα activates ERK signaling. **a**, **b** Human lung cancer cells, HCC827 (**a**) and H1650 (**b**), transfected with GFP (control), constitutively active IKKα, or kinase-dead IKKα were lysed using 1× RIPA buffer supplemented with protease and phosphatase inhibitors. Equal amounts of proteins were separated with 4–20% SDS-PAGE and transferred to polyvinyl difluoride membranes. Antigen-coated membranes were incubated overnight with primary antibodies against IKKα, phosphorylated DARPP-32 (Thr-34), total DARPP-32, phosphorylated PP1α (Thr320), total PP1α, phosphorylated ERK (Thr202/Tyr204), total ERK, and α-tubulin (loading control). **c**, **d** Vehicle (DMSO)- or calyculin A-treated human HCC827 (**c**) and H1650 (**d**) cells were lysed with 1× RIPA buffer and subjected to immunoblotting using anti-phospho PP1α (Thr320), -total PP1α, -phospho ERK (Thr202/Tyr204), -total ERK, -DARPP-32, and -α-tubulin (loading control) antibodies. Chemiluminescence signals were detected after incubating membranes with HRP-tagged secondary antibodies. Representative images from one experiment are shown, but results were validated by performing three independent biological repeats. Bar graphs at the right show quantification of the results from the three western blotting experiments. Error bars indicate SEM. *P < 0.05; ns not significant.

A seminal report suggested that the neuronal phosphoprotein DARPP-32 acts as a potent inhibitor of PP1 following phosphorylation by PKA at the Thr-34 position^24^. On the basis of this report, we hypothesized that IKKα-mediated DARPP-32 phosphorylation inhibits PP1α activity in NSCLC cells and promotes oncogenic growth by activating cell signaling pathways. To test our hypothesis, we transiently overexpressed constitutively active and kinase-dead IKKα plasmids in HCC827 and H1650 cells and performed an immunoblotting experiment with antibodies directed against phosphorylated DARPP-32 (T34). In line with our previous in vitro kinase results, we observed that expression of phosphorylated DARPP-32 increases to a greater extent in HCC827 and H1650 cell lysates overexpressing active IKKα than in GFP- or kinase-dead IKKα–expressing cell lysates (Fig. 3a, b). Phosphorylation of PP1α by cdc2 kinases in NIH-3T3 cells inhibits PP1α phosphatase activity in a cell cycle–dependent manner^38^, and phosphorylation of DARPP-32 at the T34 position leads to DARPP-32–mediated phosphorylation and inactivation of PP1α in neurons and cancer cells^24,39^. We, therefore, sought to determine the effect of IKKα expression on the levels of inactive PP1α protein in immunoblotting experiments using anti-phospho PP1α antibodies. Expression of phosphorylated (inactive) PP1α proteins increases in stable DARPP-32-overexpressed NSCLC cells upon transient expression of constitutively active IKKα cDNA plasmids compared to GFP- or kinase-dead IKKα- expression plasmids (Fig. 3a, b). As expected, transfection of constitutively active IKKα cDNA plasmids in T34A DARPP-32-overexpressed HCC827 and H1650 cells shows no increase in phosphorylated PP1α expression (Supplementary Fig. 1a, b). Collectively, our findings suggest that overexpression of IKKα leads to increased DARPP-32 phosphorylation at the T34, which inhibits PP1 phosphatase activity. To test how repression of PP1 function by the IKKα/DARPP-32 complex stimulates downstream oncogenic cell signaling, we focused on the ERK/MAPK signaling pathway because pharmacologic inhibition of PP1 activity has been reported to increase ERK activity^40^. In immunoblotting experiments, we observed an increase in the expression of phosphorylated ERK in HCC827 and H1650 cells exogenously expressing constitutively active IKKα compared to GFP- or kinase-dead IKKα- transfected cells (Fig. 3a, b), whereas phosphorylated ERK expression remains unchanged upon transient expression of constitutively active IKKα cDNA plasmids in stable T34A DARPP-32-overexpressed HCC827 and H1650 cells (Supplementary Fig. 1a, b). To validate our theory that phosphorylation of ERK protein is controlled by PP1α phosphatase, we performed western blotting experiments in HCC827 and H1650 cells treated with a pharmacological inhibitor of PP1α, calyculin A. The expression of phosphorylated (i.e., inactive) PP1α, as well as phosphorylated (i.e., activated) ERK, was higher in calyculin A–treated HCC827 and H1650 cells than in vehicle-treated cells (Fig. 3c, d). Calyculin A is also known to inhibit PP2α activity, which represents a limitation of our current studies. Future similar experiments specifically ablating PP1α using shRNA or CRISPR are warranted to definitively confirm whether phosphorylation of ERK protein is controlled by PP1α phosphatase as our initial data suggests. In summary, our results suggest that overexpression of kinase-active IKKα protein positively regulates the ERK-MAPK pathway through the DARPP-32/PP1α axis.

### IKKα controls the inhibition of PP1α phosphatase activity

**Fig. 4:**
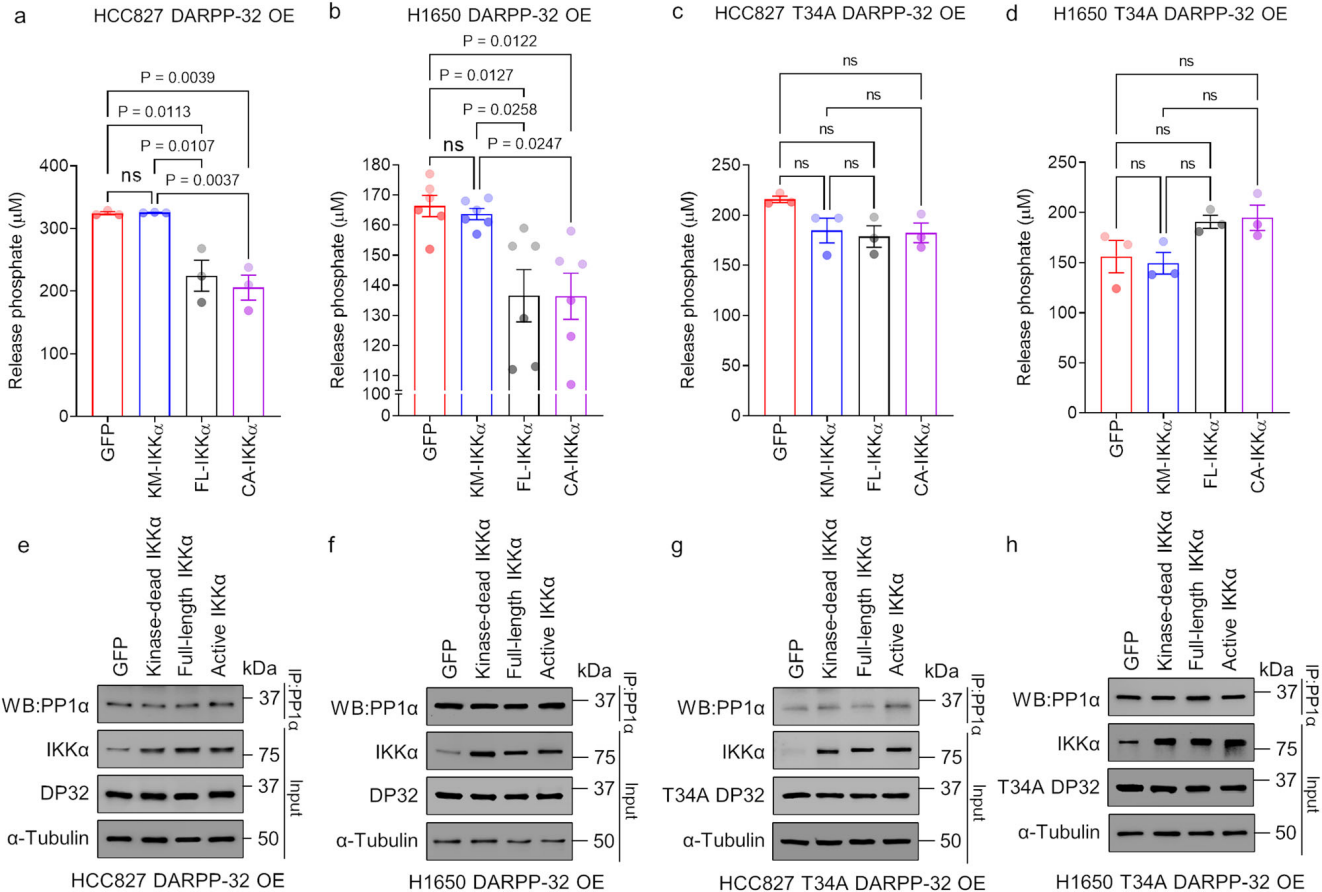
Overexpression of IKKα inhibits phosphatase activity of PP1α. **a**–**d** Human NSCLC HCC827 (**a**, **c**) and H1650 (**b**, **d**) cells transduced with retrovirus designed to overexpress either wild-type (**a**, **b**) or mutant (T34A) DARPP-32 (**c**, **d**) were transfected with GFP (control), kinase-dead (KD), full-length (FL), and constitutively active (CA) IKKα cDNAs were lysed using 1× RIPA buffer supplemented with protease inhibitors only. Equal amounts of proteins (500 ng) were immunoprecipitated using anti-PP1α antibodies. Immunoprecipitated cell lysates were subjected to in vitro phosphatase assays following incubation with either PP1α substrate or histone H1 peptide (control). Released phosphates in each reaction tube were determined by using a phosphate detection reagent. In vitro phosphatase experiments were repeated three times independently. Bar graphs represent the mean ± SEM of the three repeats, with each circle in a bar representing an independent experiment. A value of *P* ≤ 0.05 was considered significant, ns not significant, one-way ANOVA followed by Dunnett’s test. **e**–**h** Immunoprecipitated HCC827 (**e**, **g**) and H1650 (**f**, **h**) cell lysates separated with 4–20% SDS-PAGE were subjected to western blotting using anti-PP1α antibodies. Input cell lysates were blotted with antibodies against IKKα, DARPP-32, and α-tubulin (loading control).

To test our hypothesis that IKKα prevents PP1α phosphatase activity in NSCLC cells by phosphorylating DARPP-32 at Thr-34, we performed an in vitro phosphatase assay in lung adenocarcinoma cells stably overexpressing DARPP-32 protein. Briefly, kinase-dead, full-length, and constitutively active IKKα plasmids, as well as GFP-expressing control plasmids, were transiently transfected into HCC827 and H1650 cells stably overexpressing DARPP-32 protein. Endogenous PP1α was immunoprecipitated from the cell lysates and subjected to phosphatase assays. We observed decreased PP1α phosphatase activity (i.e., lower concentrations of released phosphates) in the lysates of cells overexpressing full-length or kinase-active IKKα relative to lysates of GFP-expressing cells (Fig. 4a, b). As expected, overexpression of kinase-dead IKKα in both cell lines failed to inhibit PP1α phosphatase activity (Fig. 4a, b). To further test whether IKKα blocks PP1α phosphatase activity via DARPP-32 phosphorylation at Thr-34, we stably overexpressed mutant DARPP-32 (T34A) in HCC827 and H1650 cells and repeated the in vitro phosphatase assay. As expected, no inhibition of PP1α activity was seen in cells overexpressing full-length or constitutively active IKKα in the presence of mutant DARPP-32 (Fig. 4c, d). To ensure that equal amounts of immunoprecipitated PP1α were used in the in vitro phosphatase assay, we performed immunoblotting experiments to measure the expression level of PP1α in different groups. We observed that equal amounts of PP1α were immunoprecipitated in HCC827 and H1650 cells exogenously expressing kinase-dead, full-length, or constitutively active IKKα or GFP (Fig. 4e–h). Taken together, our findings indicate that IKKα-mediated DARPP-32 phosphorylation inhibits PP1α phosphatase activity.

### Depletion of IKKα expression in tumor cells inhibits oncogenic growth advantage

**Fig. 5:**
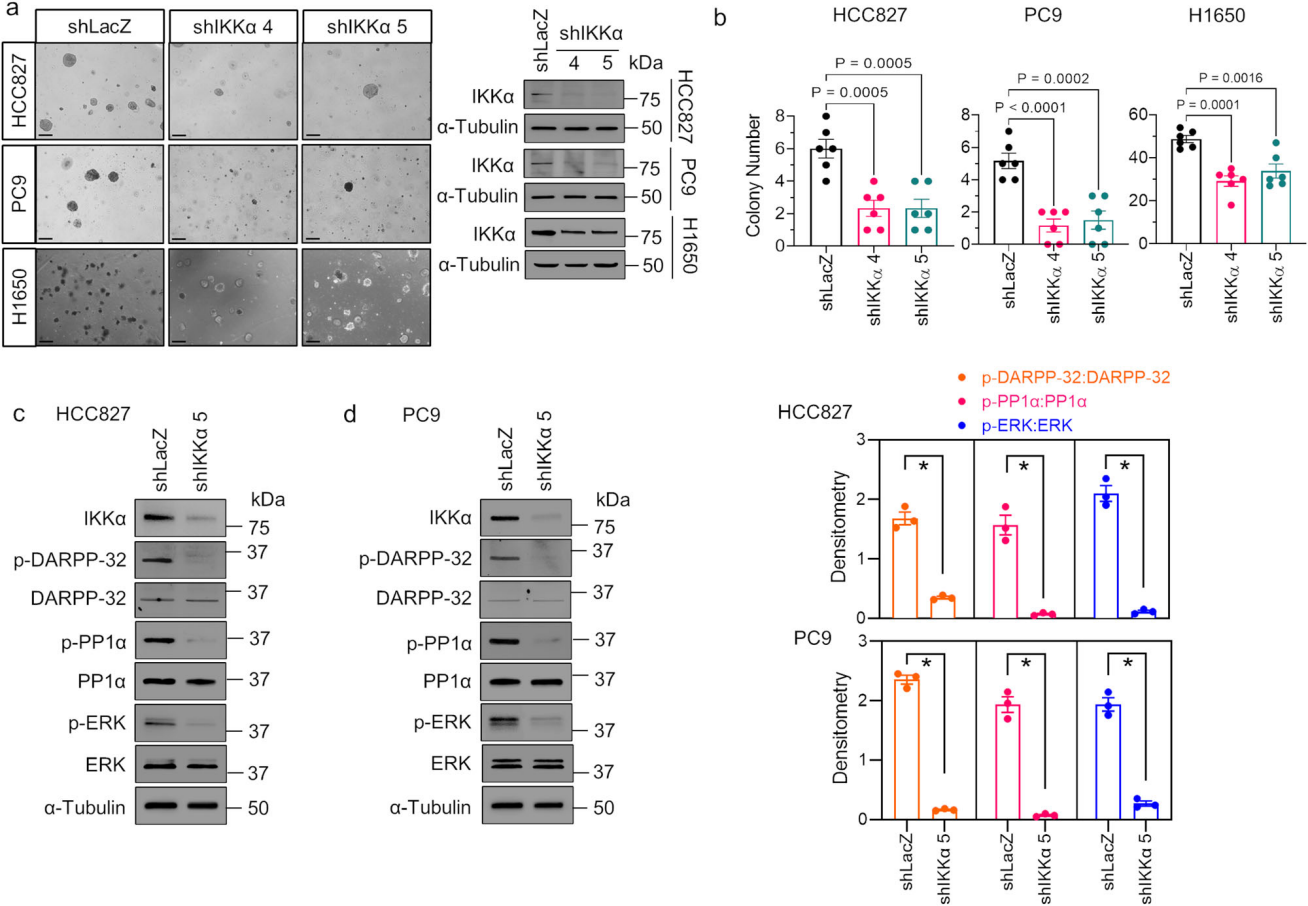
Knockdown of IKKα protein expression blocks anchorage-independent NSCLC cell growth. **a** Representative images of HCC827, PC9, and H1650 cells transduced with lentivirus encoding either LacZ shRNA or IKKα shRNAs forming colonies on soft-agar cell culture dishes 1 to 2 weeks after plating. **b** Human NSCLC HCC827, PC9, and H1650 cells transduced with lentivirus designed to silence LacZ (control) or IKKα protein expression were subjected to soft-agar colony formation assays to determine anchorage-independent cell growth. ImageJ was used to count colonies on the cell culture dishes after 1 to 2 weeks of incubation, and the number of counted colonies was plotted. Each circle on a graph represents an independent experiment. Soft-agar colony formation experiments were repeated at least six times. Error bars indicate SEM (*n* = 6). Scale bar 200 µm. A value of *P* ≤ 0.05 was considered significant, one-way ANOVA followed by Dunnett’s test. **c**, **d** Lysates from HCC827 (**c**) and PC9 (**d**) cells transduced with either LacZ shRNA or IKKα shRNA were subjected to immunoblotting with primary antibodies against IKKα, phosphorylated DARPP-32 (Thr-34), total DARPP-32, phosphorylated PP1α (Thr320), total PP1α, phosphorylated ERK (Thr202/Tyr204), total ERK, and α-tubulin (loading control). Bar graphs at the right show values obtained from the densitometric quantification of the results from three western blotting experiments. *P < 0.05; Two-tailed unpaired *t*-test.

**Fig. 6:**
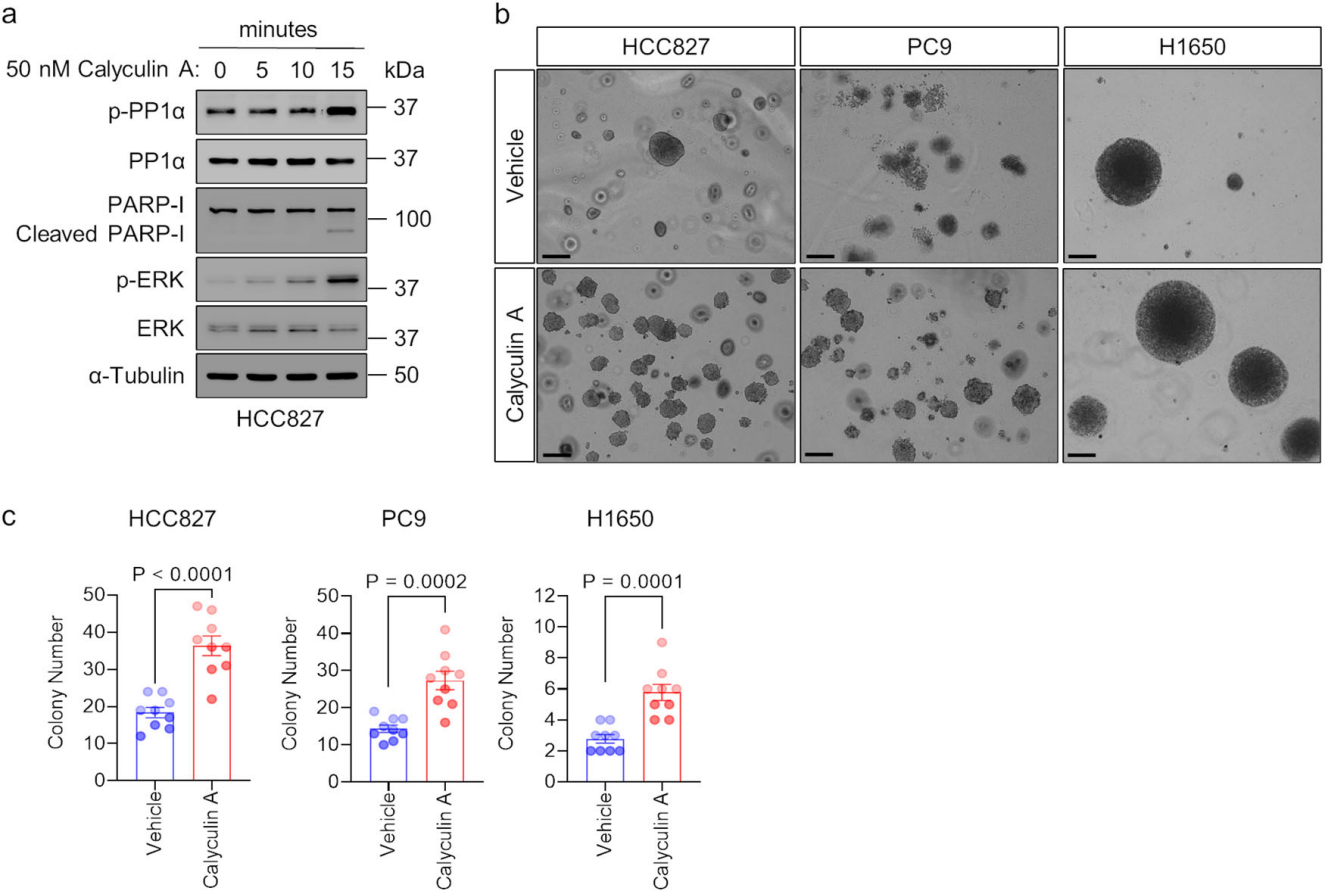
Inhibition of PP1 phosphatase activity promotes anchorage-independent lung cancer cell growth. **a** Human lung cancer HCC827 cells treated with calyculin A (50 nM) for the indicated times were lysed using 1× RIPA buffer supplemented with protease and phosphatase inhibitors. An equal amount of protein was separated with 4–20% SDS-PAGE and transferred to polyvinyl difluoride membranes. Antibody-reactive protein bands were visualized after overnight incubation of primary antibodies against phosphorylated PP1α (Thr320), total PP1α, PARP-I, phosphorylated ERK (Thr202/Tyr204), total ERK, and α-tubulin (loading control). **b** Human NSCLC HCC827, PC9, and H1650 cells were incubated with vehicle (DMSO) or calyculin A (50 nM) for 15 min prior to plating. Representative images indicate cell colonies grown on the soft-agar cell culture dishes after 2 weeks of incubation. Scale bar 200 µm. **c** Bar graphs show the average count of cell colonies observed after 2 weeks of incubation. Bar graphs indicate mean ± SEM (*n* = 9). A value of *P* ≤ 0.05 was considered significant, two-tailed unpaired *t*-test.

**Fig. 7:**
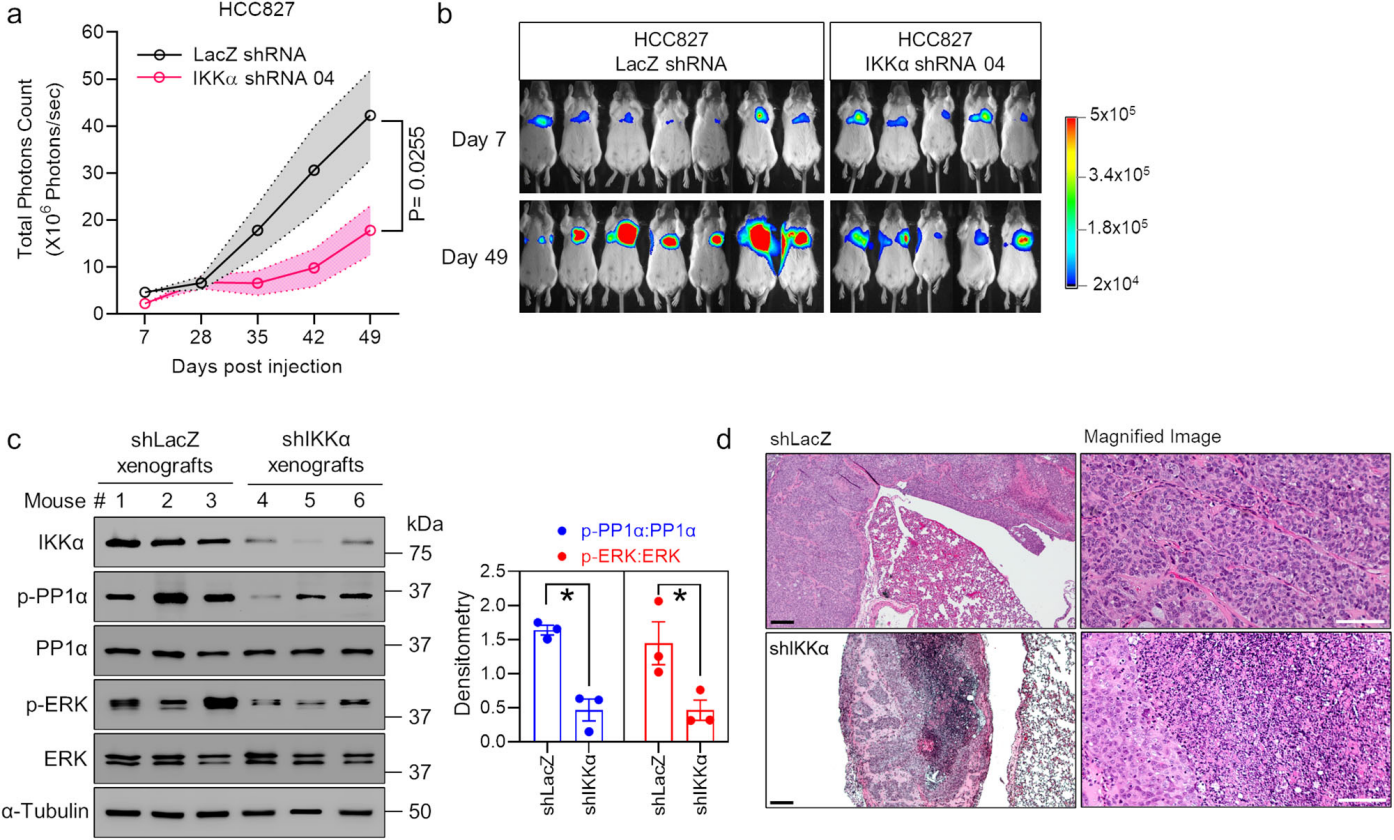
Depletion of IKKα inhibits lung tumor cell growth and proliferation in vivo. **a** Luciferase-labeled IKKα-depleted human HCC827 cells were orthotopically injected into the left thorax of SCID mice and imaged for luminescence on the indicated days. Total luminescence intensity (photon count) was calculated using molecular imaging software and plotted as a line graph. Error bars are shown as dotted lines indicating SEM. A value of *P* ≤ 0.05 was considered significant, two-way ANOVA followed by Sidak’s test. **b** Images of anesthetized mice were captured to detect luminescence signals on the indicated days. **c** Tumor tissue lysates obtained from either LacZ shRNA- or IKKα shRNA-transduced human HCC827 cells-derived xenografts were subjected to immunoblotting using primary antibodies against IKKα, phosphorylated PP1α (Thr320), total PP1α, phosphorylated ERK (Thr202/Tyr204), total ERK, and α-tubulin (loading control). Bar graphs show densitometric quantification values of the results from three western blotting experiments. *P < 0.05; Two-tailed unpaired *t*-test. **d** Overall morphological evaluation was performed on formalin‐fixed, paraffin‐embedded lung tissues (*n* = 5 mice per group) obtained from human HCC827 cells-derived lung tumor xenograft mice model using hematoxylin and eosin (H&E) dye. Scale bar 50 µm.

To test the premise that IKKα promotes oncogenic tumor growth, we first performed soft agar anchorage-independent growth assays in human lung adenocarcinoma HCC827, PC9, and H1650 cells because anchorage-independent growth is considered one of the most reliable markers of malignant transformation^41^. We observed less anchorage-independent growth (number of colonies formed on the soft-agar plates) of HCC827, PC9, and H1650 cells transduced with IKKα shRNAs relative to corresponding LacZ shRNA-transduced controls (Fig. 5a, b). We next performed immunoblotting experiments to investigate molecular mechanisms of IKKα-mediated oncogenic tumor growth. Upon knockdown of IKKα, we observed decreased expression of each of phosphorylated DARPP-32 at Thr-34 (i.e., reduced activity), -PP1α (i.e., increased activity), and -ERK (i.e., reduced activity) compared to LacZ shRNA-transduced control cells (Fig. 5c, d), suggesting that IKKα promotes anchorage-independent oncogenic lung tumor growth through regulation of the DARPP-32/PP1α/ERK cell signaling pathway. We previously showed that DARPP-32 promotes lung cancer growth through studies modulating DARPP-32 expression in human lung adenocarcinoma cells that were orthotopically xenografted into SCID mice^25^. To confirm the role of PP1α in the regulation of tumor cell growth, we performed anchorage-independent soft agar growth assays in calyculin A-pretreated HCC827, PC9, and H1650 cells. Results from immunoblotting experiments show an increase in phosphorylated PP1α (i.e., inactive) and phosphorylated ERK (i.e., activated) expression after 15 min of calyculin A treatment with a slight increase in cell death, as suggested by the detection of a small amount of cleaved PARP-I at 15 min (Fig. 6a). We observed that pharmacologic inhibition of PP1α in HCC827, PC9, and H1650 human NSCLC cells increased the number of colonies grown on soft agar, suggesting that reduced PP1α activity promotes anchorage-independent lung cancer cell growth (Fig. 6b, c). We then tested whether IKKα ablation reduces lung tumor growth in an orthotopic xenograft mouse model. Briefly, luciferase-labeled human HCC827 NSCLC cells were injected into the left thorax of anesthetized SCID mice. After the establishment of the lung tumor, mice were imaged for bioluminescence signals weekly over the course of 7 weeks. Mice challenged with IKKα-ablated HCC827 cells showed less lung tumor growth than mice administered cells transduced with control LacZ shRNA (Fig. 7a, b). Immunoblotting experiments demonstrated a reduction in the expression of phosphorylated PP1α (i.e., inactive) and phosphorylated ERK (i.e., activated) in lung tumor tissue lysates harvested from mice challenged with IKKα-ablated HCC827 lung cancer cells relative to lysates harvested from mice that were administered control LacZ shRNA-transduced HCC827 cells (Fig. 7c). We stained mouse lung tumor tissues with hematoxylin and eosin (H&E), and we observed a necrotic core within tumors derived from the mice challenged with stable IKKα-depleted HCC827 cells, whereas lung cancer tissues harvested from mice that received LacZ shRNA-transduced HCC827 cells had little to no necrosis (Fig. 7d). This finding helps explain the tumor growth retardation observed in mice challenged with stable IKKα-depleted HCC827 cells relative to controls. Taken together, our in vitro cellular studies and in vivo mouse data suggest that IKKα protein drives lung oncogenic tumor growth, and ablation of IKKα expression reduces lung cancer growth.

## Discussion

Here, we show that the kinase function of IKKα phosphorylates full-length DARPP-32 protein at the Thr-34 position, which leads to the inactivation of PP1 and subsequent activation of ERK signaling to promote lung tumor growth. The IKK complex, consisting either of IKKα, -β, and -γ kinases (canonical) or IKKα homodimers (noncanonical), has been studied in the context of inflammation and innate immunity as a regulator of interferon regulatory factors and NF-κB signaling^42–44^. Recently, it has been appreciated that IKKα and related kinases also phosphorylate proteins involved in controlling biological processes, including cell growth, metabolism, apoptosis, cell cycle, cell migration, and invasion, independent of NF-κB–regulated cell signaling pathways^43,45,46^. Overexpression of constitutively active IKKα influences the proliferation of mammary epithelium through regulation of RANK signaling in a genetically engineered mouse model^47^; thus, it is expected that aberrant IKKα expression promotes breast tumorigenesis. Indeed, Bennett et al. reported that overexpression of cytosolic IKKα protein is associated with reduced time to recurrence and worsened disease-free survival in estrogen receptor–positive breast cancer patients^48^. Additionally, the role of IKKα in promoting breast cancer growth in the presence of anti-estrogen therapy via activation of the Notch pathway has been well studied and provides a mechanism for hormone therapy resistance in an NF-κB–independent manner^49^. Recently, Dan and colleagues reported that IKKα protein activates the AKT cell signaling pathway by phosphorylating the mTOR complex 2 in cervical, prostate, lung, and pancreatic cell lines, establishing the oncogenic role of IKKα protein in promoting tumor growth^50^. Additionally, transcripts of *CHUK* (IKKα), but not *IKBKB* (IKKβ), are overexpressed in lung adenocarcinoma tissues compared with normal lung tissues^19^. A previous study by our group has shown that NSCLC patients with elevated IKKα expression have significantly reduced overall survival than those with low IKKα expression and that IKKα regulates NSCLC cell migration by forming a complex with DARPP-32 to influence the noncanonical NF-κB cell signaling pathway^25^. Here, we propose an alternative mechanism in which activated IKKα protein promotes NSCLC growth through a DARPP-32/PP1 cell signaling cascade in an NF-κB–independent manner.

DARPP-32 protein, encoded by the *PPP1R1B* gene, has been well studied in the nervous system to understand the complexity of signal transduction in neurons, especially striatal projection neurons^51^. The function of DARPP-32 in amplifying responses to many external stimuli is tightly regulated by its phosphorylation on multiple sites by different protein kinases. Notably, DARPP-32 phosphorylation at Thr-34 by PKA in response to extracellular signals has been shown to inhibit PP1 function in neurons^23,51^. Similarly, in the context of cancer, PKA has been shown to phosphorylate DARPP-32 at Thr-34 in response to Wnt-5a-mediated stimulation of cAMP^39^. It is well-established that cAMP-induced PKA activation leads to DARPP-32 phosphorylation at Thr-34, which results in the inhibition of PP1 activity. However, kinases do not regulate t-DARPP through phosphorylation of Thr-34 because the t-DARPP protein isoform lacks the first 36 amino acids that are present in the full-length DARPP-32 protein, including Thr-34.

DARPP-32 and t-DARPP promote non-small cell lung cancer growth through IKKα-dependent activation of noncanonical NF-κB2 signaling based on our previous findings showing DARPP-32 and t-DARPP each have oncogenic properties in lung cancer cells, including regulation of cell survival and migration^25^. We also showed in NSCLC^25,34^ and small cell lung cancer^26^ that overexpression of DARPP-32 or t-DARPP leads to increased ERK1/2 activation, whereas shRNA-mediated knockdown of DARPP-32 isoforms results in reduced ERK1/2 phosphorylation. Our findings presented here suggest that overexpression of kinase-active IKKα protein positively regulates the ERK pathway through the DARPP-32/PP1α axis. Specifically, we observed greater inactivation (i.e., phosphorylation) of PP1α proteins in NSCLC cells stably overexpressing exogenous DARPP-32 upon transient expression of constitutively active IKKα cDNA plasmids relative to wild-type or kinase-dead IKKα control expression vectors. Conversely, NSCLC cells stably overexpressing full-length DARPP-32 containing a T34A mutation that were transiently transfected with constitutively active IKKα cDNA did not exhibit increased phosphorylation (i.e., inactivation) of PP1α relative to the aforementioned controls. Thus, our collective findings suggest that IKKα phosphorylates DARPP-32 at the Thr-34 residue, which causes DARPP-32 to inactivate PP1α via phosphorylation, leading to activation (i.e., greater phosphorylation) of ERK1/2. Because the N-terminally truncated t-DARPP protein isoform lacks the Thr-34 residue, IKKα does not regulate t-DARPP through the phosphorylation of Thr-34. Upregulation of t-DARPP leads to increased activation of ERK signaling^25,26,34^; however, the molecular mechanism(s) by which t-DARPP activates ERK signaling in lung cancer are not well understood. The strong presence of t-DARPP protein phosphorylated at Thr-39 (equivalent to full-length DARPP-32 Thr-75) in our assays warrants future investigation to identify the molecular mechanism(s) of t-DARPP regulation by upstream kinases in NSCLC cells. t-DARPP may be phosphorylated by IKKα and/or other kinases at sites other than Thr-39 in t-DARPP (i.e., Thr-75 in full-length DARPP-32). An in vitro kinase experiment using radiolabeled ATP may shed light on whether IKKα phosphorylates t-DARPP protein at other sites.

Recently, it was shown that breast cancer patients with elevated DARPP-32 expression but low PP1 expression have worse overall survival than those with low expression of DARPP-32^52^, suggesting a strong inverse correlation between PP1 and DARPP-32 proteins in patient outcome. This supports the notion that DARPP-32–mediated inactivation of PP1 functions via phosphorylation leads to increased activation of kinases involved in oncogenic signaling pathways. Moreover, PKA protein expression in breast tumor tissues shows a strong correlation with DARPP-32 and PP1 protein expression, warranting further investigation to understand the molecular mechanism in regulating breast tumorigenesis^52^. In a separate study, Hansen et al. reported that PKA protein activated by Wnt-5a ligands regulates breast cancer cell migration by phosphorylating DARPP-32 at Thr-34 in a PP1/CREB-dependent manner^39^. In line with this observation, our prior report suggests DARPP-32 promotes NSCLC cell migration^25^, and our current study provides strong evidence that NSCLC cell growth is regulated by the IKKα/DARPP-32/PP1/ERK cell signaling pathway. Overexpression of t-DARPP has been shown to confer resistance to trastuzumab, a HER2-targeted monoclonal antibody, via activation of PKA and PI3K/AKT cell signaling in HER2^+^ breast cancer cells^53,54^. A molecular mechanism has recently been identified in which t-DARPP phosphorylated by CDK-1 and -5 activates PKA kinase function by forming a direct complex with PKA regulatory subunits in breast cancer cells overexpressing t-DARPP^32,33^.

The catalytic subunit of PP1, a major protein phosphatase in human cells composed of α, β, and γ subunits, regulates critical cellular processes, including cell cycle progression, apoptosis, and metabolism by catalyzing dephosphorylation of a wide range of proteins^55^. The role of PP1 as a tumor suppressor or oncogene depends on the type of cancer, the stage of cancer progression, and the regulatory proteins that interact with PP1. The pathways are further complicated because both oncogenes and tumor suppressor proteins are known substrates of PP1, and dephosphorylation events can activate or downregulate downstream cell signaling pathways^56^. Therefore, detailed mechanistic insight is needed to understand the role of PP1 in lung cancer. The complex of PP1 with the leucine-rich repeat protein SHOC2 promotes tumor growth in a subset of *KRAS*-mutant NSCLC cell lines by dephosphorylating a critical inhibitory site on RAF kinases, resulting in RAF-ERK pathway activation. Moreover, genetic inhibition of SHOC2 suppresses tumor development in autochthonous murine *Kras*-driven lung cancer models^57^. In contrast, activated PP1, upon forming a complex with protein 4.1 N, a neuronal homolog of the erythrocyte membrane cytoskeletal protein 4.1, inhibits lung tumor progression by suppressing the JNK cell signaling pathway^58^. Our results suggest that PP1-mediated dephosphorylation of ERK is inhibited by the DARPP-32/PP1 complex, which in turn promotes lung tumor growth by increasing ERK activity. Increased activation of ERK is associated with elevated oncogenic potential due to the central position of ERK downstream of several oncogenic growth signaling pathways.

Manipulation of PP1 activity has long been considered a potential approach to treating cancer because of the involvement of PP1 in several cancer-related cellular processes. The small-molecule inhibitors calyculin A and okadaic acid have been used to mitigate PP1 and PP2A activity, thereby impairing the progression of hormone therapy-resistant prostate cancer by stimulating cell death^59^. However, PP1 small-molecule inhibitors have unwanted cellular toxicity because PP1 is involved in a broad range of cellular processes. Moreover, the homology of the active sites among different phosphatases contributes to the limited efficacy of these inhibitors in treating cancer. Therefore, targeting PP1 complexes, instead of focusing on the catalytic sites of PP1, is a promising solution to suppress sustained growth and survival in cancer.

Recent findings of interesting, novel phosphorylation substrates of IKK family kinases, including DARPP-32 in this study, expand current knowledge of critical biological and disease-related mechanisms. To comprehensively understand the function of these pleiotropic kinases, further experiments are needed to assess the roles of IKK family members in regulating phosphorylation-dependent substrates in different settings and diseases. In this regard, it will be important to see whether DARPP-32 phosphorylation is regulated by IKKα protein in the presence of anticancer agents routinely used in the clinic to treat lung cancers. Another critical question—which upstream kinases regulate IKKα activation—warrants further investigation because EGFR and KRAS are highly mutated in lung cancer patients^60^. Importantly, the results we report here were primarily based on studies using three EGFR-mutated NSCLC cell lines, H1650, HCC827, and PC9. KRAS is not mutated in any of these three cell lines, so testing whether ERK signaling is activated via DARPP-32-mediated inhibition of PP1 activity in the context of KRAS-mutated NSCLC warrants future investigation. Targeting IKK and IKK-related kinases with the small-molecule IKK inhibitors SAR-113945 and MLN-0415 has shown encouraging results in preclinical studies, although they failed to meet the primary endpoints of a phase 2 clinical trial and the safety profile of a phase 1 clinical trial, respectively^61^. Because NF-κB functions in many different systems, targeting IKKα and IKK-related kinases to treat disease, including cancers, can result in unpredictable adverse events. Therefore, the development of more selective, isoform-specific, non-ATP-competitive inhibitors against IKK family kinases to use in combination therapies and/or as part of a targeted delivery approach is required, particularly in cancers that aberrantly express IKKα protein.

## Methods

### Cell lines and inhibitors

Human NSCLC cell lines A549 (*KRAS*^G12S^ and *STK11*^mut^) and H1650 (*EGFR*^ΔE746-A750^ and *TP53*^deletion^), as well as a transformed human embryonic kidney epithelial cell line, HEK-293T, were purchased from the American Type Culture Collection. The epidermal growth factor receptor (EGFR)-mutated human NSCLC cell lines HCC827 (*EGFR*^ΔE746-A750^ and *TP53*^deletion^), PC9 (*EGFR*^AMP^, *EGFR*^ΔE746-A750^, *TP53*^R248Q^, and *CDKN2A*^G67V^) and H1975 (*EGFR*^L858R+T790M^ and *TP53*^R273H^) were kindly provided by Dr. Pasi A. Jänne at the Dana-Farber Cancer Institute^62^, Dr. Aaron N. Hata at Massachusetts General Hospital^63^, and Dr. Anthony C. Faber at Virginia Commonwealth University^64^, respectively. Dulbecco’s modified Eagle’s medium (DMEM; Corning, Cat no. 10-013-CV) supplemented with 10% fetal bovine serum (FBS; Millipore, Cat no. TMS-013-B) was used to grow HEK-293T cells. Human NSCLC cell lines A549, H1650, HCC827, PC9, and H1975 were maintained in Roswell Park Memorial Institute (RPMI)-1640 medium (Corning, Cat no. 10-040-CV) supplemented with 10% FBS (Millipore), 1% penicillin/streptomycin antibiotics (Corning, Cat no. 30-002-CI), and 25 µg/mL plasmocin prophylactic (Invivogen, Cat no. ant-mpp). All cell lines were routinely authenticated via morphologic inspection and tested negative for mycoplasma contamination. A serine/threonine protein phosphatase inhibitor, calyculin A, purchased from Cell Signaling Technology (CST, Cat no. 9902), was used to mitigate PP1α function.

### Generation of stable cell lines

Human full-length DARPP-32, t-DARPP, and mutant DARPP-32 (T34A) cDNAs cloned into the pcDNA3.1 vector were kindly provided by Dr. Wael El-Rifai at the University of Miami Health System^65^. The FLAG-tagged coding sequence of DARPP-32, t-DARPP, and DARPP-32 T34A were subcloned into a retroviral (pMMP) vector. Retrovirus containing FLAG-tagged full-length DARPP-32, t-DARPP, and mutant DARPP-32 cDNAs were prepared by following a previously described procedure^25,34^. Briefly, 5 µg of the cDNA plasmids (pMMP-DARPP-32, pMMP-t-DARPP, pMMP-T34A DARPP-32, or corresponding control pMMP-LacZ), 1.5 µg of pMD.MLV.gag.pol packaging plasmid DNA, and 0.5 µg of pMD.2 G envelope plasmid DNA were used to transfect 10 cm dishes of 293 T cells after mixing with 300 µl of EC buffer (Qiagen), 32 µl of Enhancer (Qiagen), and 30 µl of Effectene (Qiagen, Cat no. 301425). Media was replaced 16 h post-transfection. The supernatant containing retroviral particles was collected at 48 and 72 h post-transfection. Viral supernatant was filtered through 0.45-micron sterile filters, concentrated via Retro-X Concentrator (Clontech, Cat no. 631455), resuspended in 1 ml RPMI-1640 media, aliquoted, and stored at −80 °C until used. NSCLC cells seeded at a density of 3 × 10^5^ cells per 10-cm cell culture dish were transduced with 1 mL retrovirus diluted in 5 mL fresh medium supplemented with 10 µg/mL polybrene solution (Millipore, Cat no. TR-1003-G). Cells were used for subsequent experiments 48 h after transduction. The pMMP plasmid, its corresponding control pMMP-LacZ vector, the pMD.MLV.gag.pol packaging plasmid, and the pMD.2 G envelope plasmid were kindly provided by Dr. Debabrata Mukhopadhyay at Mayo Clinic, Jacksonville, FL.

Lentiviral vectors (pLKO.1) designed to silence IKKα (shIKKα #4: GCAAATGAGGAACAGGGCAAT; shIKKα #5: GCGTGCCATTGATCTATATAA) and LacZ as a control (shLacZ: CCAACGTGACCTATCCCATTA) were purchased from Sigma. Briefly, 5 µg of the lentiviral plasmids, along with their corresponding packaging plasmids (similar to the retroviral transfection method), were transfected into human 293 T cells using the Effectene transfection reagent (Qiagen) as described above. Fresh complete growth medium was added to cell culture plates at 16 h post-transfection. Viral supernatant was collected at 48 and 72 h post-transfection, filtered through 0.45-micron sterile filters, concentrated using Lenti-X concentrators (Clontech, Cat no. 631231), and used immediately to transduce HCC827 and PC9 lung cancer cell lines, as reported previously^66^. Transduced cells were incubated in a medium containing puromycin (Sigma, Cat no. P8833) for 72 h to select stable IKKα knockdown cells.

Retroviruses containing the luciferase gene were prepared in 293 T cells as described previously^26^. Briefly, the retrovirus-based firefly luciferase expression vector (MSCV IRES Luciferase) along with packaging plasmid (gag/pol) and envelope expressing plasmid (pCMV-VSV-G) from Addgene were co-transfected into 293 T cells. The MSCV IRES Luciferase plasmid, gag/pol vector, and pCMV-VSV-G plasmid were gifts from Scott Lowe (Addgene plasmid #18760), Tannishtha Reya (Addgene plasmid #14887)^67^, and Bob Weinberg (Addgene plasmid #8454)^68^, respectively. Retroviral particles were collected after 48 and 72 h of transfection, filtered through 0.45-micron sterile filters, concentrated via Retro-X Concentrator (Clontech, Cat no. 631455), resuspended in complete RPMI-1640 medium, aliquoted, and stored at −80 °C until used.

To generate luciferase-labeled stable human NSCLC cells, 0.6 × 10^6^ cells were plated in 6 cm cell culture dishes and incubated at 37 °C overnight. On the next day, human NSCLC cells were transduced by adding a mixture of 3 ml complete cell culture growth media, 3.5 µl Polybrene (10 µg/ml; Millipore), and 500 µl concentrated virus. Cells were selected using 500 µg/ml of Hygromycin (Sigma, Cat no. H7772) every 2–3 days until the corresponding plates of Hygromycin-treated control (i.e., not transduced) cells had died. Luciferase-labeled stable human NSCLC cells were used to determine tumor growth in orthotopic murine models.

### Antibodies

Primary antibodies (1 µg/µl) identifying two different phosphorylated sites on DARPP-32 (T34: cat no. 12438; dilution 1:1000; and T75: cat no. 2301; dilution 1:1000), phosphorylated PP1α (T320; cat no. 2581; dilution 1:1000), IKKα (cat no. 2682; dilution 1:1000), phosphorylated p44/42 MAPK (T202/Y204; cat no. 4370; dilution 1:1000), and total p44/42 MAPK (cat no. 4695; dilution 1:1000) were purchased from CST. Antibodies (200 µg/ml) against DARPP-32 (cat no. sc-398360; dilution 1:200), PP1 (cat no. sc-7482; dilution 1:100), and α-tubulin (cat no. sc-5286; dilution 1:500) were obtained from Santa Cruz Biotechnology. Horseradish peroxidase (HRP)–conjugated secondary antibodies against the heavy chains of anti-rabbit (cat no.: 7074; dilution 1:5000) and anti-mouse (cat no.: 7076; dilution 1:5000) IgG were purchased from CST.

### Plasmids

Expression vectors of full-length (#15467) and kinase-dead (#15468) mouse IKKα, as well as constitutively kinase-active (#64608) human IKKα, were purchased from Addgene. Briefly, the investigators constructed a full-length IKKα in-frame with DNA encoding an N-terminal FLAG epitope in pCR-3 vectors^69^. Kinase-dead IKKα (K44A) was generated from full-length IKKα expression plasmids by using a site-directed mutagenesis kit^69^. Expression vectors for V5 epitope–tagged constitutively kinase-active IKKα (S176E, S180E) were constructed in destination/expression vector pcw107 via the Gateway cloning system^70^. When overexpressed, constitutively kinase-active IKKα (S176E, S180E) plasmid constitutively activates the NF-ĸB pathway in a ligand-independent manner. Expression plasmids (pCMV) encoding GFP used as transfection controls were kindly shared by Dr. Georgiy Aslanidi at The Hormel Institute, University of Minnesota.

### Immunoblotting

Radioimmunoprecipitation assay (RIPA; 0.5 M Tris-HCl, pH 7.4; 1.5 M NaCl, 2.5% deoxycholic acid, 10% NP-40, 10 mM EDTA) buffer (Millipore, Cat no. 20-188) supplemented with protease inhibitor cocktail (Roche, Cat no. 5892970001) and phosphatase inhibitors (Millipore, Cat no. 524629) were used to lyse human NSCLC cells on ice. Protein was quantified using Quick start^TM^ Bradford 1X dye reagent (Bio-Rad, Cat no. 5000205) using a standard curve created with different concentrations of BSA. Equal amounts of cell lysates were separated via 4–20% gradient SDS-PAGE (Bio-Rad; Cat no. 4568094) and transferred to polyvinyl difluoride membranes (Millipore, Cat no. IPVH00010). Prior to primary antibody incubation, membranes were cut into pieces based on the location of the pre-stained blue molecular weight marker (Bio-Rad, Cat no. 1610393) such that multiple membranes derived from the same immunoblotting gel could be stained with different antibodies detecting differently sized proteins. These membranes were then incubated in Tris-buffered saline (50 mM Tris-Cl, pH 7.6; 150 mM NaCl; GrowCells, Cat no. 75800-902) containing 5% bovine serum albumin (Sigma, Cat no. A7906) at room temperature for 1 h. Incubation of diluted primary and secondary antibodies was carried out overnight at 4 °C and for 2 h at room temperature, respectively. Chemiluminescence substrate (Thermo Fisher Scientific, Cat no. PI34580) was used to detect antibody-reactive protein bands in the membranes, and signals were captured electronically using an ImageQuant™ LAS 4000 instrument (GE Healthcare). To detect protein bands of similar molecular weight, we performed multiple concurrent immunoblotting experiments using equal aliquots of the same cell lysates run on different gels and/or membranes. All immunoblots derived from concurrent immunoblotting experiments were processed in parallel. Images of uncropped and unprocessed scans of the immunoblots are included in Supplementary Figs. 2–21.

### Purification of DARPP-32 isoforms

Human lung adenocarcinoma A549, HCC827, PC9, and H1975 cells stably overexpressing FLAG-tagged DARPP-32 or t-DARPP proteins were grown to 95–100% confluency in 150-mm cell culture plates. Cells were lysed on ice using 1 ml of 1 × lysis buffer (50 mM Tris-HCl, pH 7.4, with 150 mM NaCl, 1 mM EDTA, and 1% Triton X-100) supplemented with protease inhibitor cocktail (Roche). For each cell line, lysates were pooled from five plates and incubated overnight with anti-FLAG M2 agarose (Sigma, Cat no. A2220) on a rotating platform at 4 °C. Positively selected FLAG fusion proteins were collected by centrifugation at 1000×*g* for 5 min and washed with TBS. Protein elution was carried out under native conditions by incubation with 200 μl 3X FLAG peptide (GLPBIO, Cat no.GP10149FS) at a concentration of 150 ng/μl in TBS for 30 min at 4 °C and then collected following centrifugation for 30 s at 5000×*g*. The elution process was repeated twice and a total of 400 μl eluted FLAG fusion protein was concentrated to 40 μl using 0.5 mL Ultracel^®^ 30 K membrane (Millipore, Cat no. UFC503008). Protein was quantified using Quick start^TM^ Bradford 1X dye reagent (Bio-Rad, Cat no. 5000205) using a standard curve created with different concentrations of BSA. Eluted proteins were run on 4–20% polyacrylamide gels in denatured conditions and visualized following Coomassie blue (Bio-Rad, Cat no. 1610786) staining.

### In vitro kinase assay

Human DARPP-32 isoforms purified from NSCLC cells were incubated with kinase-activated human IKKα protein (SignalChem, Cat no. C51-10G-10) in 50 μl reaction volume for in vitro kinase assays by following previously described methods^71^. Briefly, 3 μg purified DARPP-32 isoforms as well as 5 μl ATP (New England Biolabs, Cat no. N0440) in kinase dilution buffer III (5 mM MOPS, pH 7.2; 2.5 mM β-glycerol-phosphate, 5 mM MgCl_2_, 1 mM EGTA, 0.4 mM EDTA, 50 ng/µl BSA; SignalChem, Cat no. K23-09-05) were incubated with 1 μg commercially available human IKKα protein (SignalChem) for 30 min at 30 °C, then at 95 °C for 5 min, in which 1 × Laemmli sample buffer (Bio-Rad, Cat no. 1610747) supplemented with 10% β-mercaptoethanol (Bio-Rad, Cat no. 1610710) was added to stop the kinase reaction. Phosphorylation of DARPP-32 by kinase-activated human IKKα protein was validated via immunoblotting using monoclonal primary antibodies against phosphorylated DARPP-32 (T34 and T75, CST).

### Transient transfection

Human NSCLC cell lines, HCC827 or H1650, were plated in 6-well cell culture plates at a concentration of 2 × 10^5^ cells per well. Cells were washed with PBS (Corning, Cat no. 1610747) on the following day prior to transfection, and a complete RPMI-1640 medium (Corning) was added to each well. Based on the protocols from the manufacturer, 2.5 µg of plasmid DNA and 5 µl P3000 reagent (Invitrogen, Cat no. L3000001) diluted in OPTI-MEM medium (Gibco, Cat no. 31985062) were incubated with 10 µl Lipofectamine-3000 transfection reagent (Invitrogen, Cat no. L3000001) for 15 min at room temperature. The DNA:Lipofectamine mixture was then added to each well in a dropwise manner and incubated for 48 h.

### Immunoprecipitation

Human NSCLC cell lines at a concentration of 3 × 10^6^ cells per 100-mm cell culture dish transiently transfected with either control GFP or one of three different IKKα plasmids were lysed in RIPA buffer (Millipore) supplemented with protease inhibitors (Roche). The concentration of harvested cell lysates was measured by using the Bradford reagent (Bio-Rad, Cat no. 5000205). Anti-PP1α antibody (2 µg; SCBT, Cat no. sc-7482) was added to the supplied spin column (Catch and Release Immunoprecipitation Kit; cat no. 17-500; Millipore) along with the cell lysates (500 µg) to immunoprecipitate the proteins following the manufacturer’s protocol. The eluted proteins in their native form were subsequently used to perform the in vitro phosphatase assay.

### In vitro phosphatase assay

The in vitro phosphatase assay was performed in accordance with the manufacturer’s protocol. Briefly, 5 μl PP1α substrates (GRPRTS[p]SFAEG; SignalChem, Cat no. P50-58) or 5 μl control histone H1 peptides (GGGPATP-KKAKKL-COOH; SignalChem, Cat no. H10-58) diluted in phosphatase dilution buffer II (50 mM Imidazole, pH 7.2, 0.2% 2-mercaptoethanol, 65 ng/µl BSA; SignalChem, Cat no. P22-09) was incubated for 15 min at 37 °C with human PP1α protein in its native form immunoprecipitated from human NSCLC cells at a final volume of 30 μl. The amount of free phosphate molecules generated by the reaction was colorimetrically quantified with a Phosphate Assay Kit (Abcam, Cat no. ab65622). The amount of released phosphate was determined from a standard curve generated after plotting the absorbance value against increasing known concentrations of free phosphate molecules.

### Soft-agar colony formation assay

Five milliliters of complete RPMI-1640 medium (Corning) containing 0.75% melted agar (Sigma, Cat no. A9045) was added to 60-mm cell culture dishes to create a bottom layer. Cells of the human NSCLC lines HCC827, PC9, and H1650 transduced with lentivirus encoding LacZ shRNA (control) or IKKα shRNAs were suspended in complete RPMI-1640 medium containing 0.36% melted agar and were plated on top of the bottom layer at a concentration of 2.5 × 10^4^ cells per dish. After 1 (H1650) to 2 weeks (HCC827 and PC9) of incubation, images of colonies that had grown on the soft-agar cell culture plates were captured using a 4× Plan S-Apo 0.16 NA objective on an EVOS FL cell imaging system (Thermo Fisher Scientific). The colonies were counted by using ImageJ software and plotted by using GraphPad Prism 9 software. Similarly, 3 × 10^4^ HCC827, PC9, and 1 × 10^4^ H1650 cells were first incubated with either vehicle (DMSO) or calyculin A (50 nM) for 15 min. Cells were then resuspended in fresh RPMI-1640 medium containing 0.36% melted agar and plated on the soft-agar cell culture plates. After 2 weeks of incubation, images were captured and analyzed using ImageJ software.

### In vivo orthotopic lung cancer model

Six- to eight-week-old pathogen-free SCID/NCr mice were purchased from Charles River Laboratories. Mice were allowed one week to acclimate to their surroundings, then bred, maintained under specific-pathogen-free conditions in a temperature-controlled room with alternating 12 h light/dark cycles, and fed a standard diet in accordance with protocols approved by the University of Minnesota Institutional Animal Care and Use Committee. For each mouse, luciferase-labeled human HCC827 lung cancer cells (1 × 10^6^) transduced with either LacZ shRNA (control) or IKKα shRNAs were suspended in 80 μl PBS and Matrigel (Corning, Cat no. 354248). The cells were then orthotopically injected in the right thoracic cavity of 8- to 12-week-old male and female mice and allowed to establish tumors over 1 week. Luminescence images of mice were taken weekly over 7 weeks using an In Vivo Xtreme xenogen imaging system (Bruker). The luciferase intensity (total photon count) of each mouse was calculated using Bruker molecular imaging software and plotted over time in GraphPad Prism 9 software.

### Statistics

Statistically significant differences between multiple groups (greater than 2) were determined by performing a one-way analysis of variance (ANOVA) followed by Dunnett’s test. To compare differences between two groups, two-tailed unpaired t-tests were performed. Statistically significant differences in tumor growth over time between the two groups in the mouse experiments were determined with two-way ANOVA followed by Sidak’s test. Values of *P* ≤ 0.05 were considered significant. Data were expressed as mean ± SEM of at least three independent experiments.

### Reporting summary

Further information on research design is available in the Nature Research Reporting Summary linked to this article.

## Supplementary information

REPORTING SUMMARY

Supplementary Figures

## Data Availability

The authors declare that the data supporting the findings of this study are available within the article and its supplementary information. Any other associated data supporting the findings of this study are available from the corresponding author upon request.
