# Supplementary material for: IKKα promotes lung adenocarcinoma growth through ERK signaling activation via DARPP-32-mediated inhibition of PP1 activity
Source: NPJ Precis Oncol. 2023 Mar 25;7:33. doi: 10.1038/s41698-023-00370-3 (PMC10039943; doi:10.1038/s41698-023-00370-3)
Supplement: Supplementary file 1 — REPORTING SUMMARY [file 41698_2023_370_MOESM1_ESM.pdf]

nature portfolio

Corresponding author(s): Luke H. Hoeppner & Sk. Kayum Alam

Last updated by author(s): Nov 7, 2022

Reporting Summary

Nature Portfolio wishes to improve the reproducibility of the work that we publish. This form provides structure for consistency and transparency in reporting. For further information on Nature Portfolio policies, see our Editorial Policies and the Editorial Policy Checklist.

Statistics

For all statistical analyses, confirm that the following items are present in the figure legend, table legend, main text, or Methods section.

n/a	Confirmed	
☐	☒	The exact sample size (*n*) for each experimental group/condition, given as a discrete number and unit of measurement
☐	☒	A statement on whether measurements were taken from distinct samples or whether the same sample was measured repeatedly
☐	☒	The statistical test(s) used AND whether they are one- or two-sided<br>*Only common tests should be described solely by name; describe more complex techniques in the Methods section.*
☒	☐	A description of all covariates tested
☐	☒	A description of any assumptions or corrections, such as tests of normality and adjustment for multiple comparisons
☐	☒	A full description of the statistical parameters including central tendency (e.g. means) or other basic estimates (e.g. regression coefficient) AND variation (e.g. standard deviation) or associated estimates of uncertainty (e.g. confidence intervals)
☐	☒	For null hypothesis testing, the test statistic (e.g. *F*, *t*, *r*) with confidence intervals, effect sizes, degrees of freedom and *P* value noted<br>*Give P values as exact values whenever suitable.*
☒	☐	For Bayesian analysis, information on the choice of priors and Markov chain Monte Carlo settings
☒	☐	For hierarchical and complex designs, identification of the appropriate level for tests and full reporting of outcomes
☒	☐	Estimates of effect sizes (e.g. Cohen's *d*, Pearson's *r*), indicating how they were calculated

Our web collection on statistics for biologists contains articles on many of the points above.

Software and code

Policy information about availability of computer code

Data collection	Living Image Software by Perkin Elmer
Data analysis	GraphPad Prism version 9

For manuscripts utilizing custom algorithms or software that are central to the research but not yet described in published literature, software must be made available to editors and reviewers. We strongly encourage code deposition in a community repository (e.g. GitHub). See the Nature Portfolio guidelines for submitting code & software for further information.

Data

Policy information about availability of data

All manuscripts must include a data availability statement. This statement should provide the following information, where applicable:

- Accession codes, unique identifiers, or web links for publicly available datasets
- A description of any restrictions on data availability
- For clinical datasets or third party data, please ensure that the statement adheres to our policy

The authors declare that the data supporting the findings of this study are available within the article and its supplementary information.

Human research participants

Policy information about studies involving human research participants and Sex and Gender in Research.

Reporting on sex and gender	Human research participants has not been relevant in this study.
Population characteristics	Human research participants has not been relevant in this study.
Recruitment	Human research participants has not been recruited in this study.
Ethics oversight	No study approval is required because human research participants has not been relevant in this study.

Note that full information on the approval of the study protocol must also be provided in the manuscript.

Field-specific reporting

Please select the one below that is the best fit for your research. If you are not sure, read the appropriate sections before making your selection.

☒ Life sciences ☐ Behavioural & social sciences ☐ Ecological, evolutionary & environmental sciences

For a reference copy of the document with all sections, see nature.com/documents/nr-reporting-summary-flat.pdf

Life sciences study design

All studies must disclose on these points even when the disclosure is negative.

Sample size	Cell culture: We used standard, previously reported cell culture sample sizes to report our in vitro findings.<br>Animal studies: Sample sizes were chosen based on published literature and our preliminary studies so as to provide sufficient power for statistical comparison (where appropriate).
Data exclusions	No data has been removed in this study.
Replication	Each experiment was replicated at least three times.
Randomization	Male and Female mice were assigned randomly to experimental and control groups.
Blinding	The investigators were not blinded during data collection.

Reporting for specific materials, systems and methods

We require information from authors about some types of materials, experimental systems and methods used in many studies. Here, indicate whether each material, system or method listed is relevant to your study. If you are not sure if a list item applies to your research, read the appropriate section before selecting a response.

Materials & experimental systems

n/a	Involved in the study	
☐	☒	Antibodies
☐	☒	Eukaryotic cell lines
☒	☐	Palaeontology and archaeology
☐	☒	Animals and other organisms
☒	☐	Clinical data
☒	☐	Dual use research of concern

Methods

n/a	Involved in the study	
☒	☐	ChIP-seq
☒	☐	Flow cytometry
☒	☐	MRI-based neuroimaging

Antibodies

Antibodies used	All antibodies were purchased from commercial suppliers and their unique catalogue numbers have been described in the Methods section of the manuscript (lines 361-369).
Validation	Validation of each primary antibody for the species and application can be found in the manufacturer's data sheet.

Eukaryotic cell lines

Policy information about cell lines and Sex and Gender in Research

Cell line source(s)	Cell line sources have been throughly described in the Methods section of the manuscript (lines 326-333).
Authentication	All cell lines were certified by the indicated cell bank and routinely authenticated by morphologic inspection.
Mycoplasma contamination	Cell lines were tested to ensure they were free of Mycoplasma contamination and routinely treated with Plasmocin to prevent Mycoplasma contamination from occurring.
Commonly misidentified lines (See ICLAC register)	No commonly misidentified cell lines were used in this study.

Animals and other research organisms

Policy information about studies involving animals; ARRIVE guidelines recommended for reporting animal research, and Sex and Gender in Research

Laboratory animals	Animals used for the in vivo studies: Six- to eight-week-old pathogen-free SCID/NCr mice were purchased from Charles River Laboratories.
Wild animals	The study did not involve wild animals.
Reporting on sex	This study involves both Male and Female mice.
Field-collected samples	The study did not involve samples collected from the field.
Ethics oversight	All animal studies were performed in accordance with protocols approved by the University of Minnesota Institutional Animal Care and Use Committee.

Note that full information on the approval of the study protocol must also be provided in the manuscript.
