# Supplementary material for: IKKα promotes lung adenocarcinoma growth through ERK signaling activation via DARPP-32-mediated inhibition of PP1 activity
Source: NPJ Precis Oncol. 2023 Mar 25;7:33. doi: 10.1038/s41698-023-00370-3 (PMC10039943; doi:10.1038/s41698-023-00370-3)

Supplementary Figures

Content	Page number
Supplementary Figure 1	1-2
Supplementary Figure 2	3
Supplementary Figure 3	4
Supplementary Figure 4	5
Supplementary Figure 5	6
Supplementary Figure 6	7
Supplementary Figure 7	8
Supplementary Figure 8	9-10
Supplementary Figure 9	11-12
Supplementary Figure 10	13-14
Supplementary Figure 11	15
Supplementary Figure 12	16
Supplementary Figure 13	17
Supplementary Figure 14	18
Supplementary Figure 15	19
Supplementary Figure 16	20
Supplementary Figure 17	21-22
Supplementary Figure 18	23-24
Supplementary Figure 19	25-26
Supplementary Figure 20	27-28
Supplementary Figure 21	29-30

Supplementary Figure 1

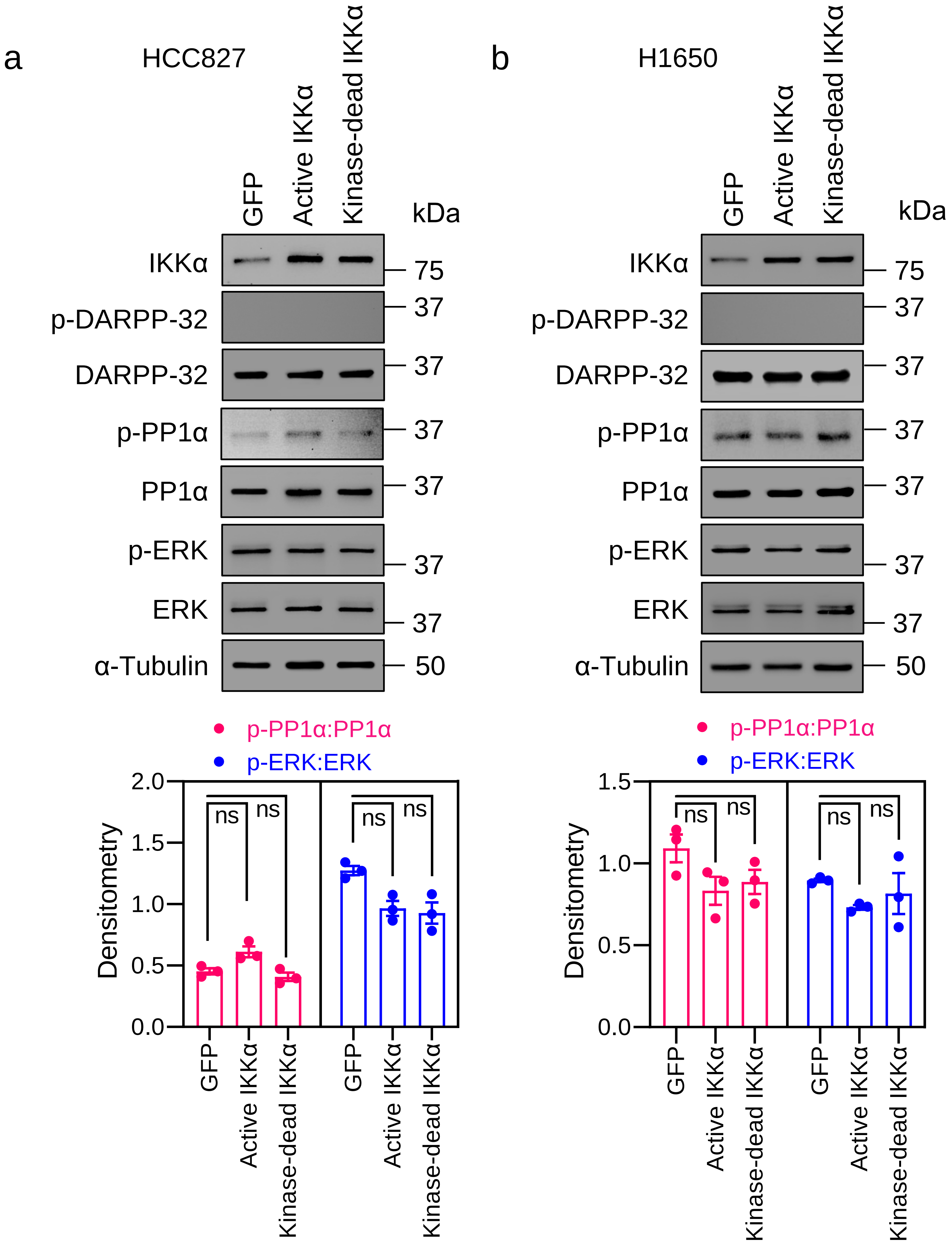

Supplementary Figure 1: Inhibition of PP1α activity is controlled by DARPP-32 phosphorylation at Thr-34 position. a-b. Cell lysates from HCC827 (a) and PC9 (b) cells transduced with retrovirus designed to overexpress mutant (T34A) DARPP-32 were subjected to western blotting using anti- IKKα, -phospho DARPP-32 (Thr34), -DARPP-32, -phospho PP1α (Thr320), -PP1α, -phospho ERK (Thr202/Tyr204), -ERK, and -α-tubulin (loading control) antibodies. Values from densitometric quantification of three independent immunoblotting experiments were plotted as bar graphs in the bottom and error bars indicate SEM. ns, not significant, one-way ANOVA followed by Dunnett's multiple testing.

Supplementary Figure 2: Full-sized scans of immunoblots in Figure 1a

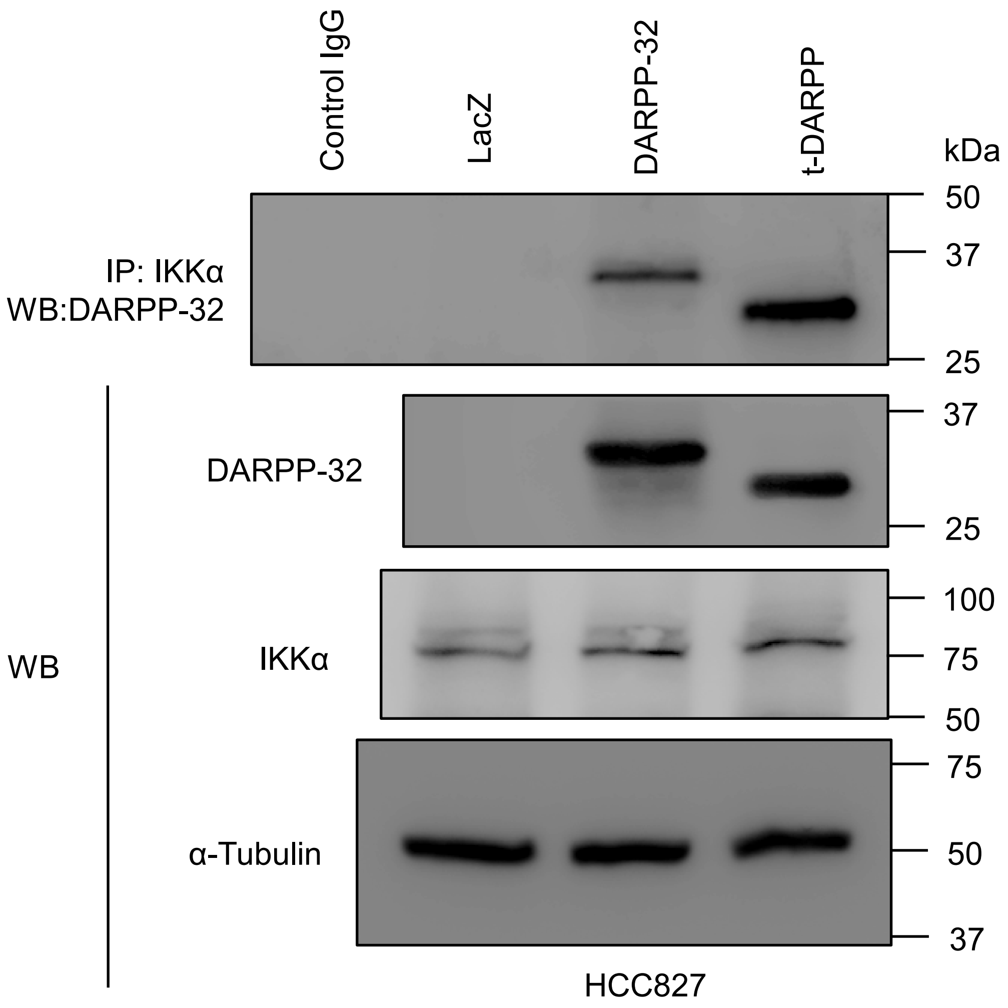

Supplementary Figure 3: Full-sized scans of immunoblots in Figure 1b

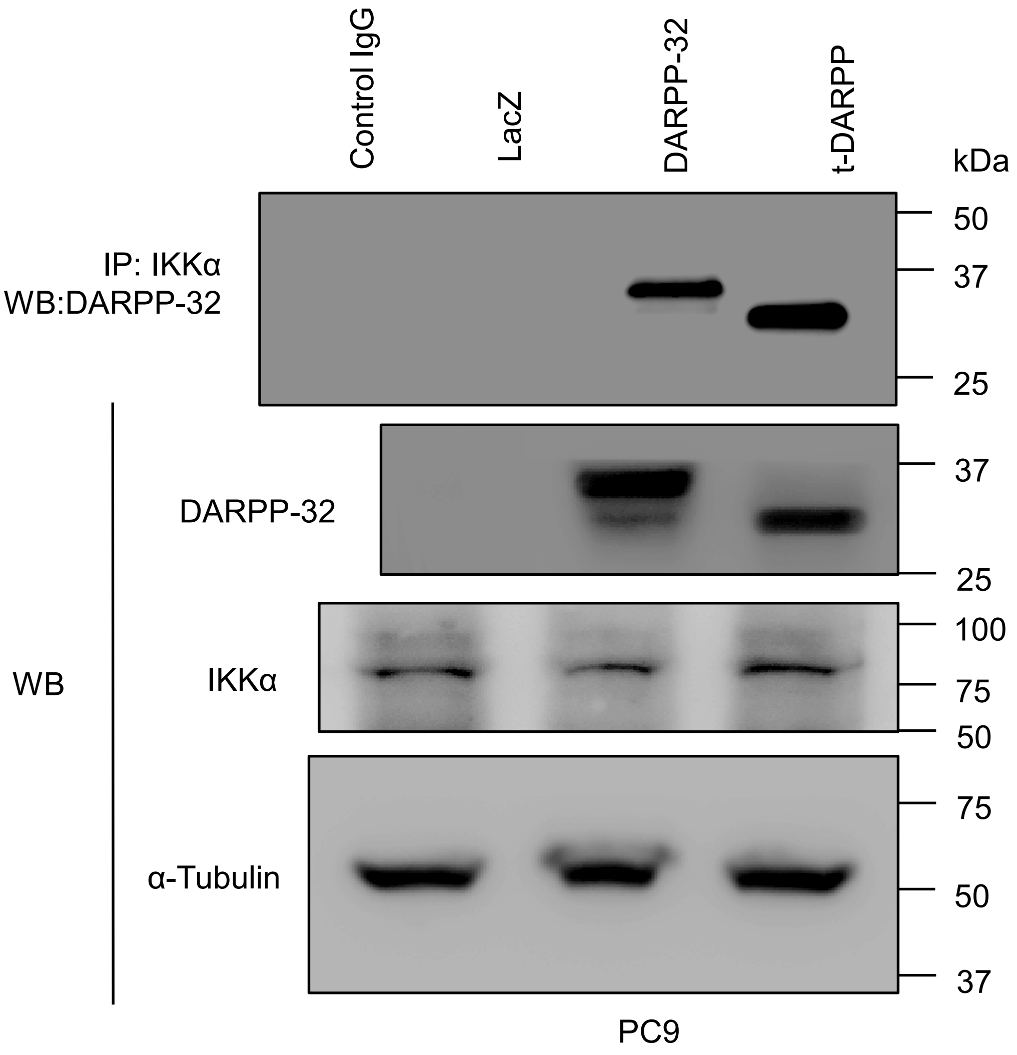

Supplementary Figure 4: Full-sized scans of immunoblots in Figure 1c

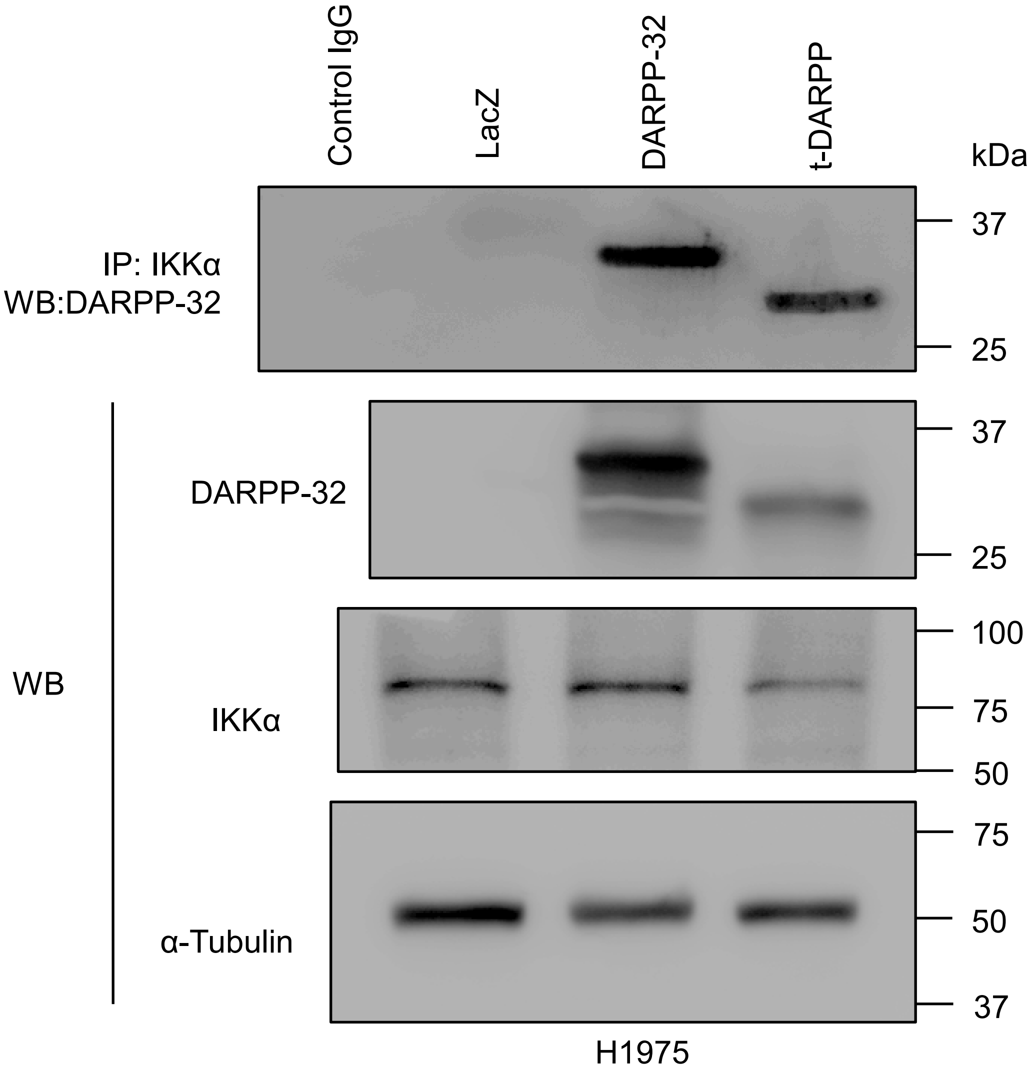

Supplementary Figure 5: Full-sized scans of immunoblots in Figure 2a

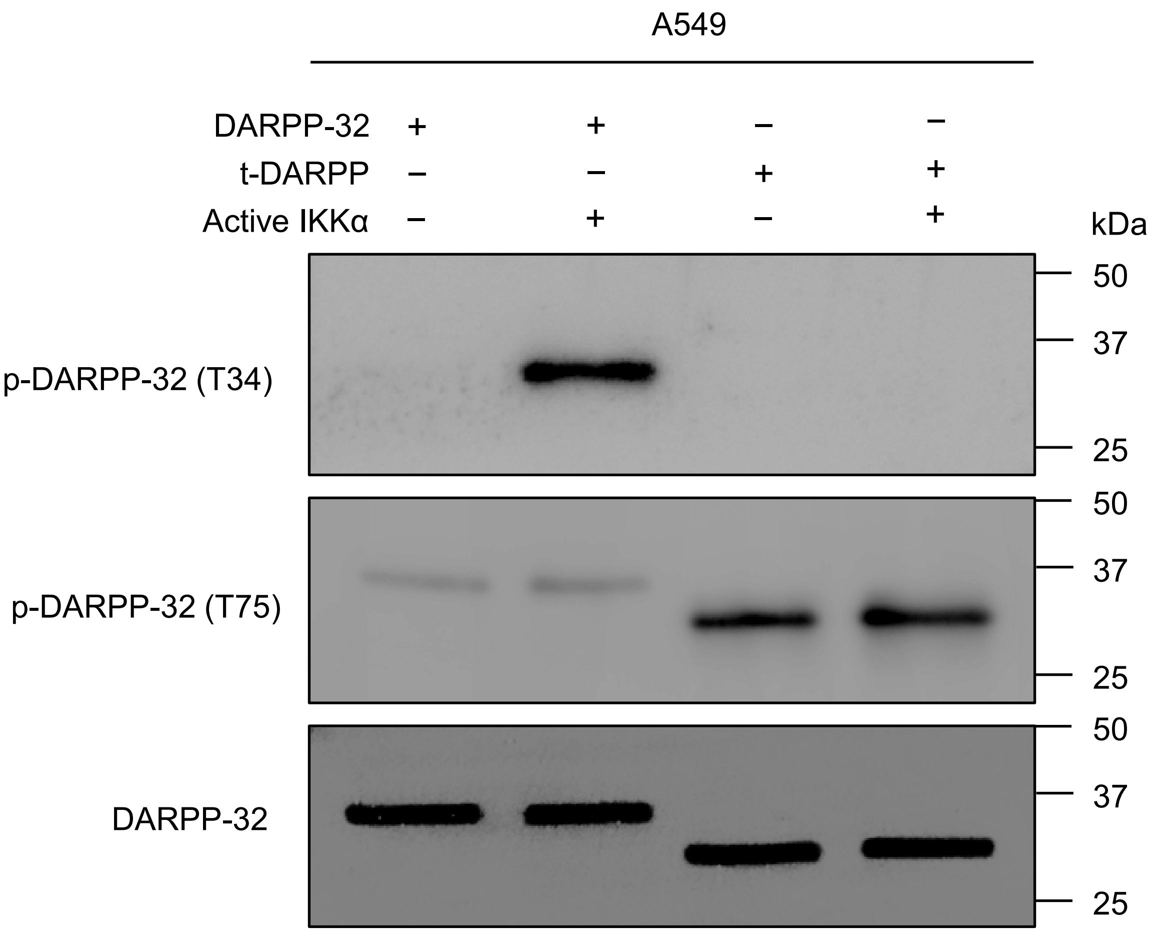

Supplementary Figure 6: Full-sized scans of immunoblots in Figure 2b

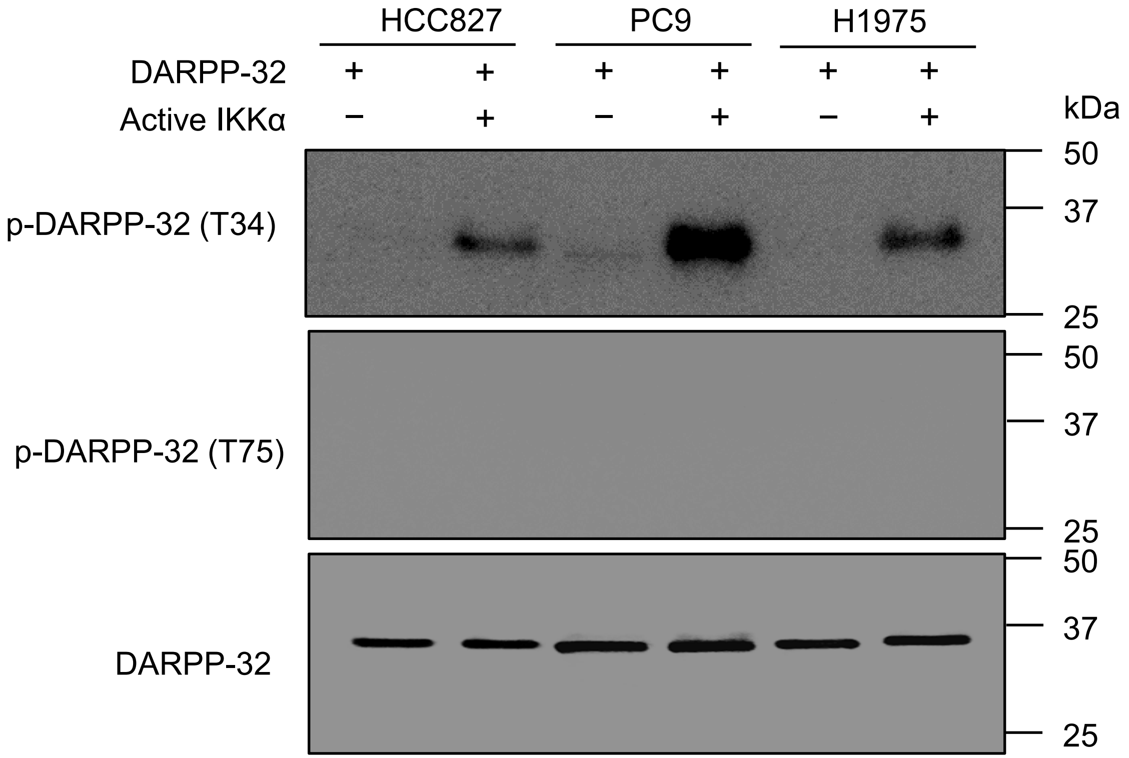

Supplementary Figure 7: Full-sized scans of immunoblots in Figure 2c

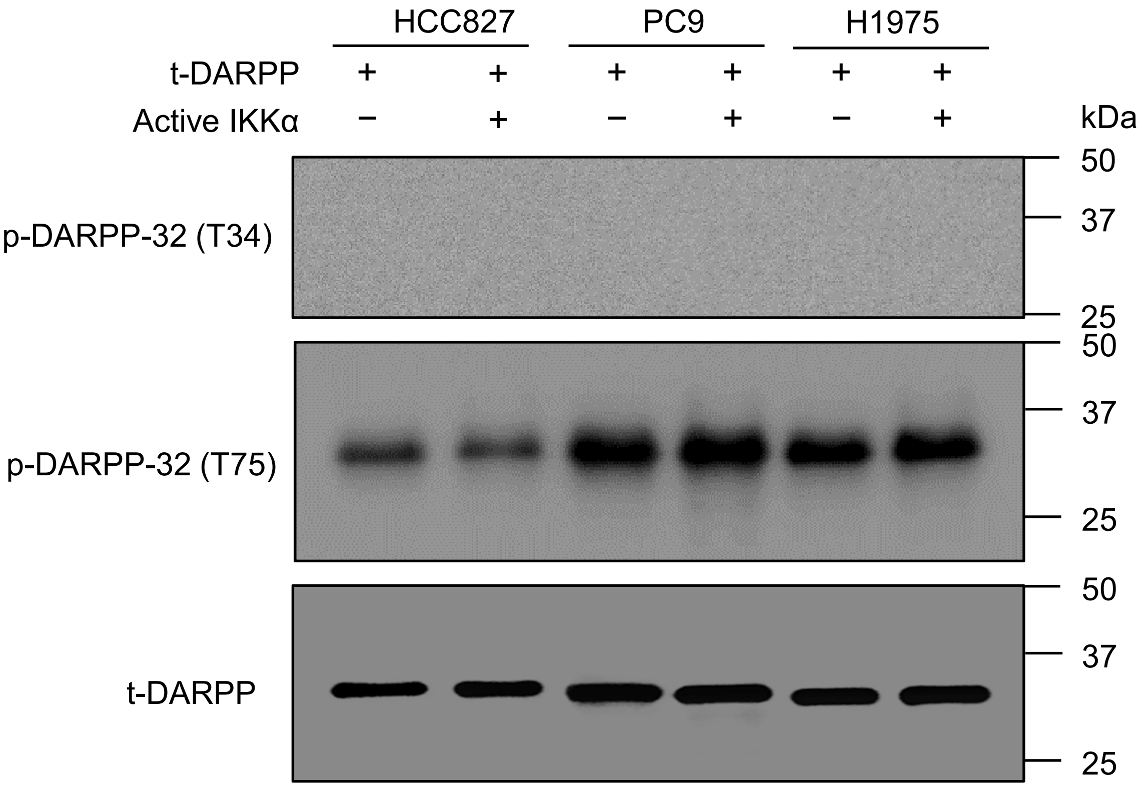

Supplementary Figure 8: Full-sized scans of immunoblots in Figure 3a

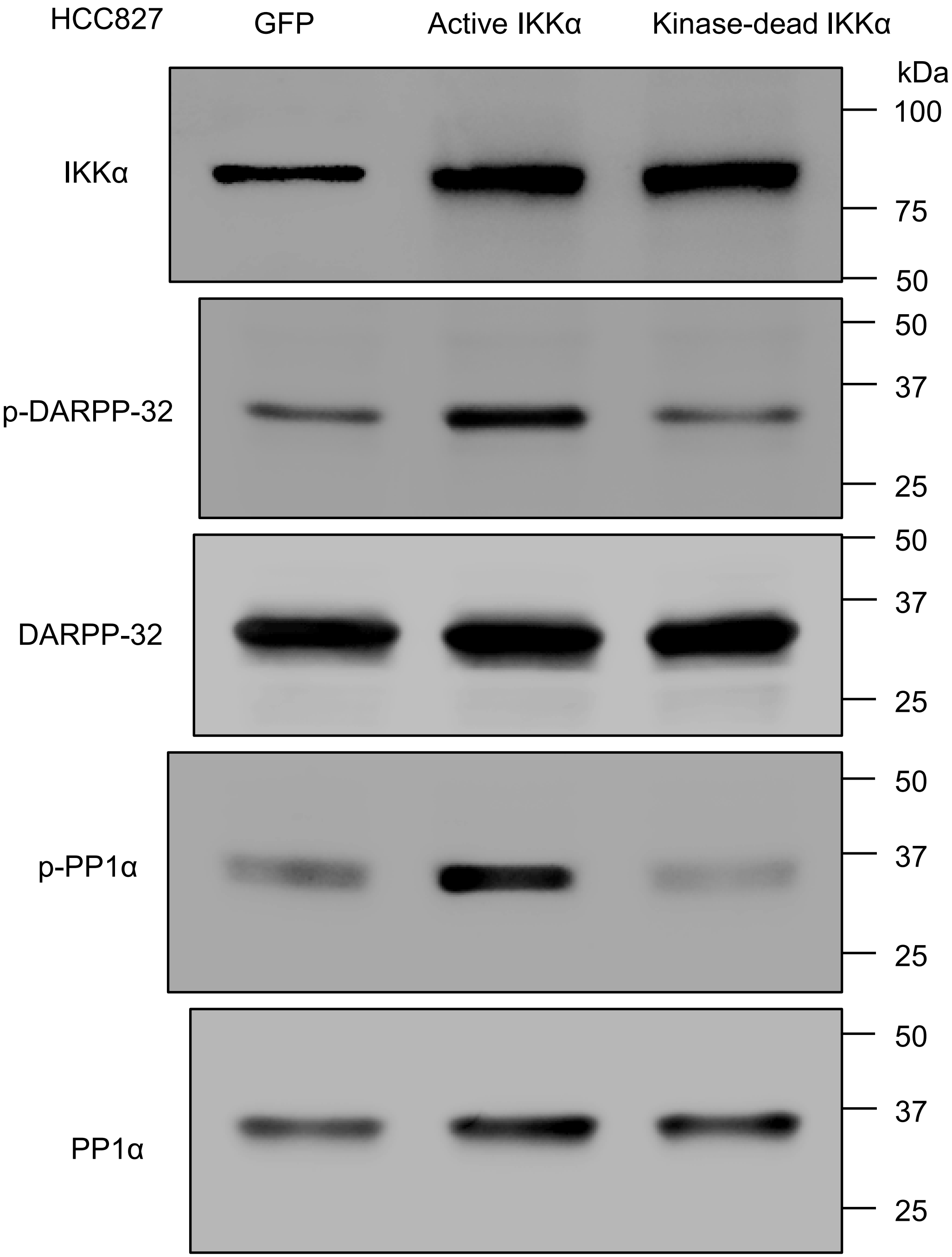

Supplementary Figure 8-continue: Full-sized scans of immunoblots in Figure 3a

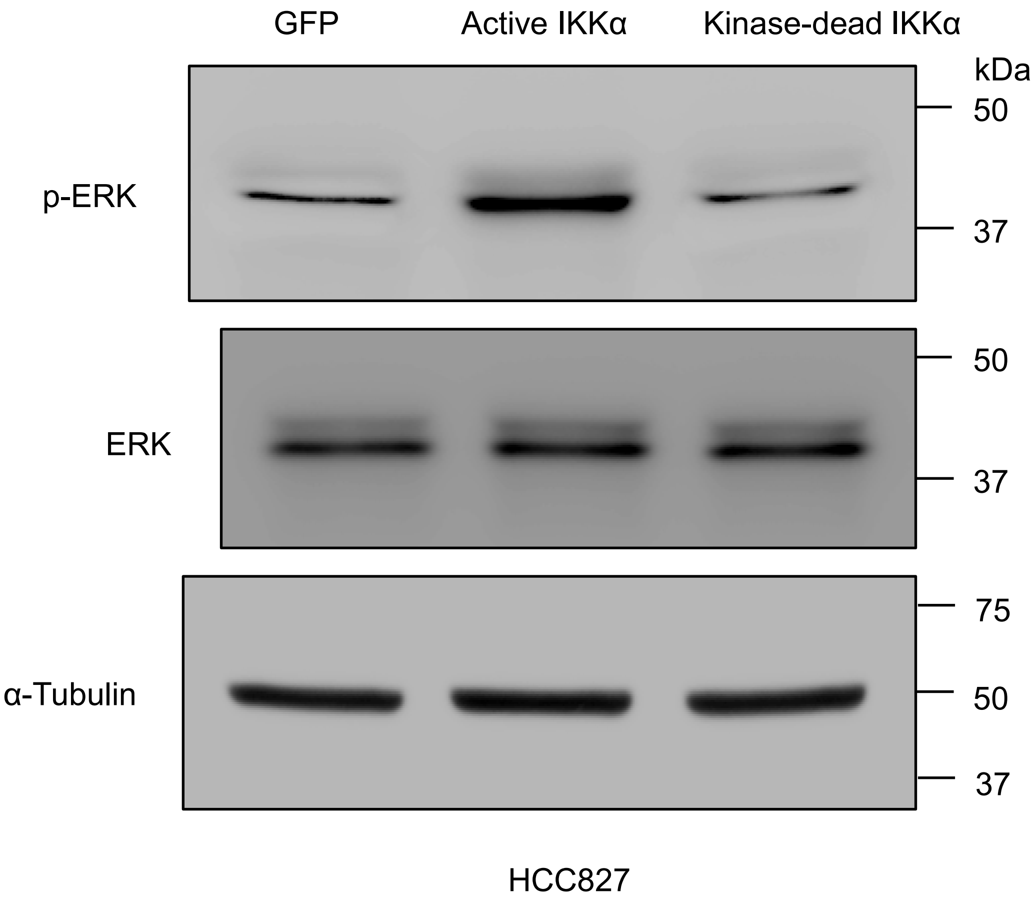

Supplementary Figure 9: Full-sized scans of immunoblots in Figure 3b

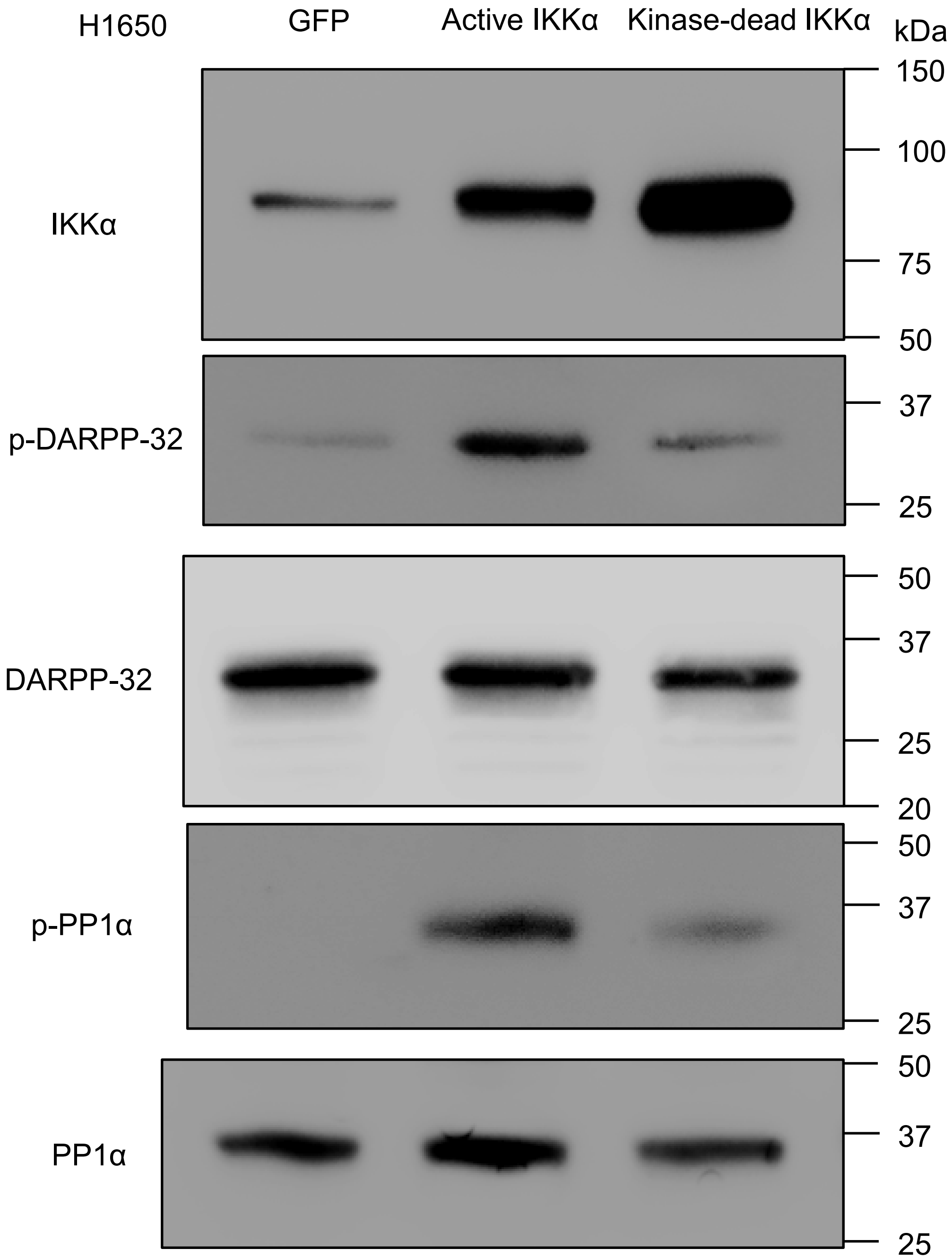

Supplementary Figure 9-continue: Full-sized scans of immunoblots in Figure 3b

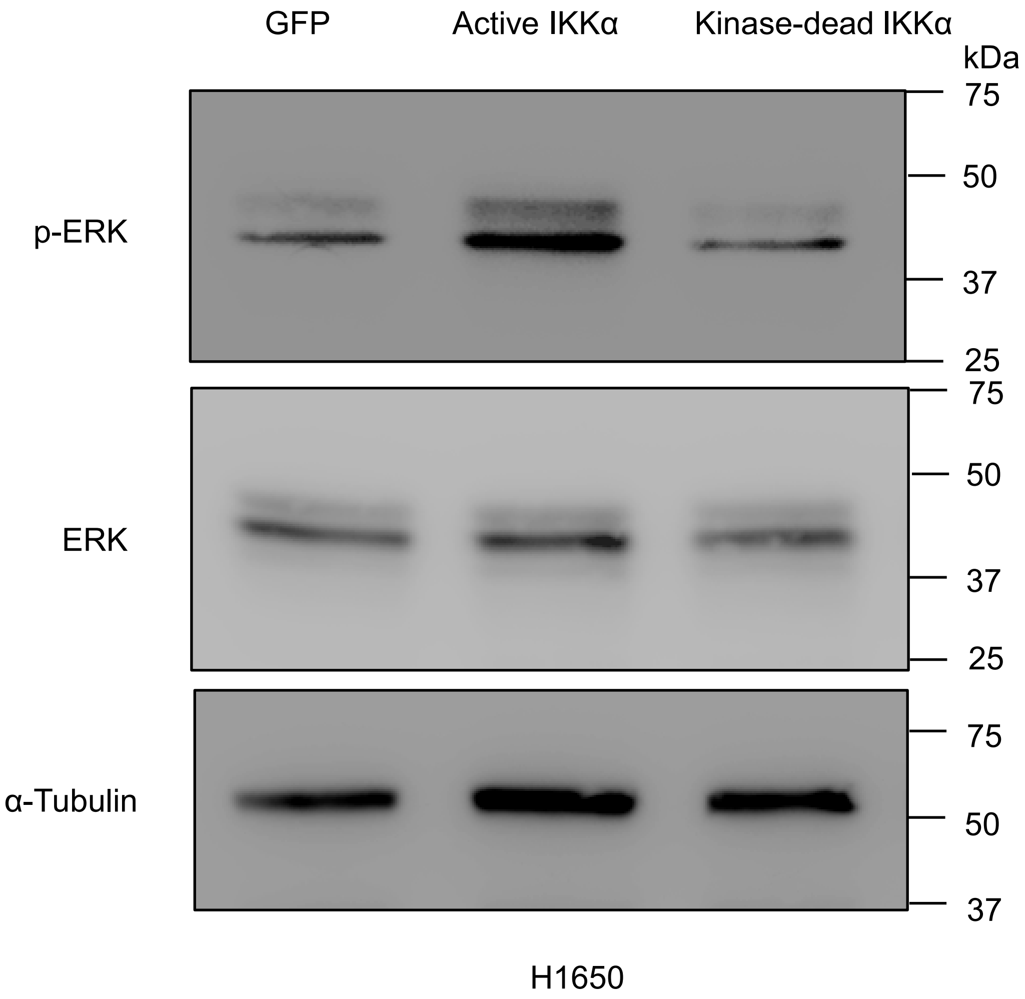

Supplementary Figure 10: Full-sized scans of immunoblots in Figure 3c

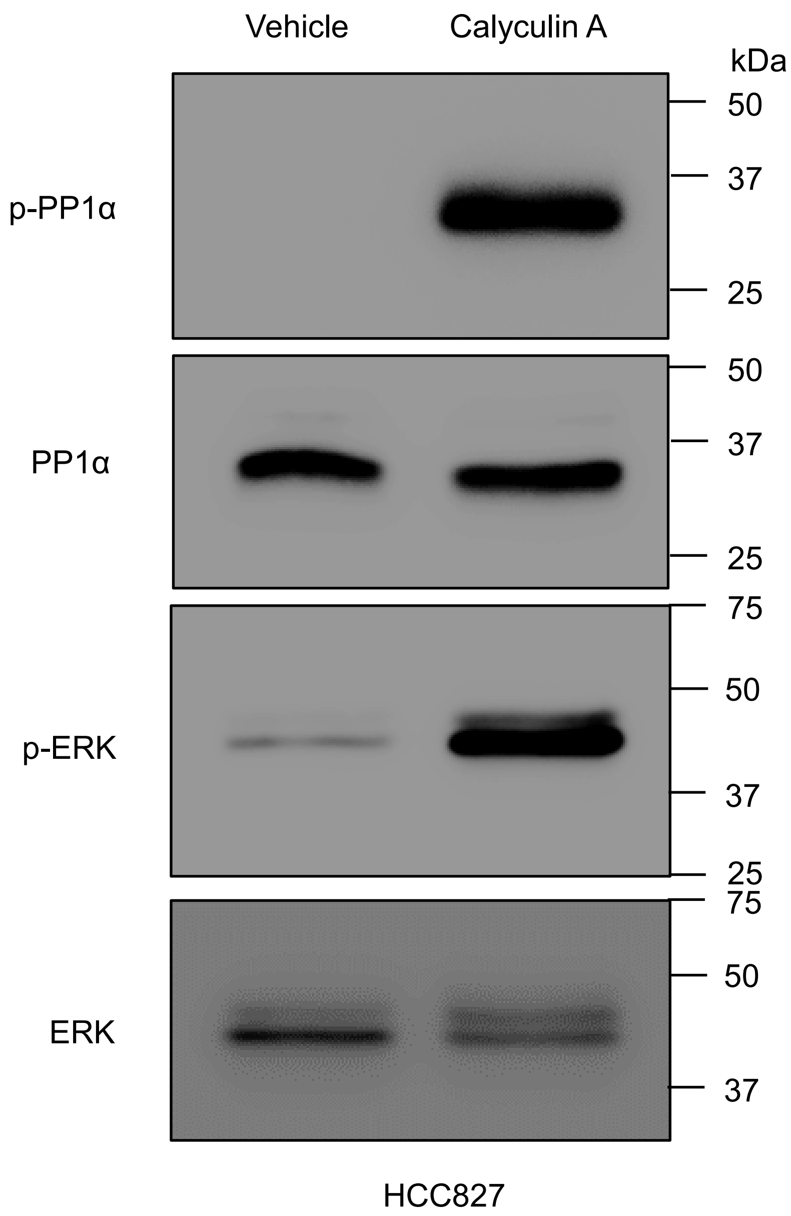

Supplementary Figure 10-continue: Full-sized scans of immunoblots in Figure 3c

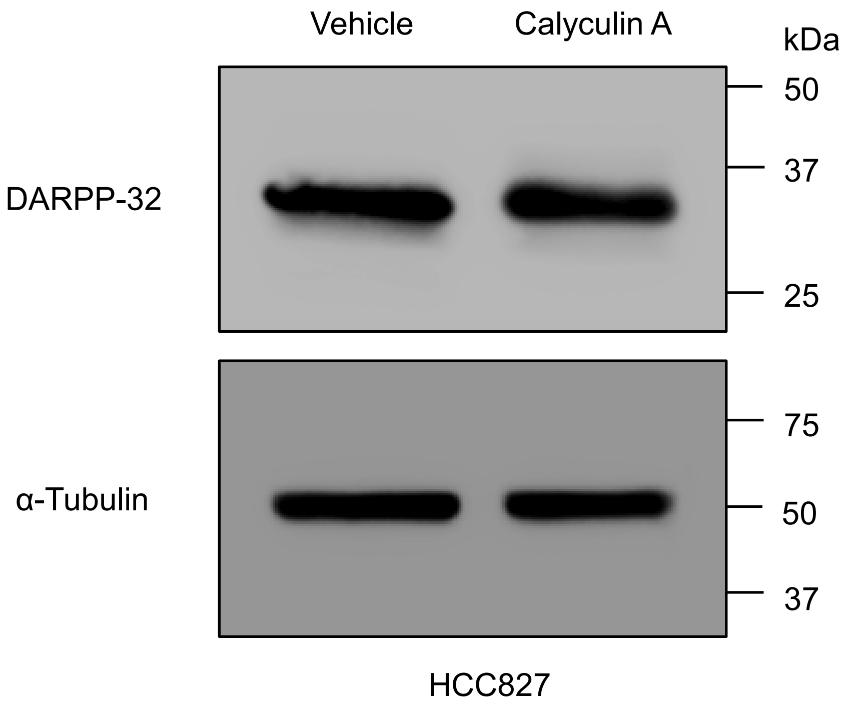

Supplementary Figure 11: Full-sized scans of immunoblots in Figure 3d

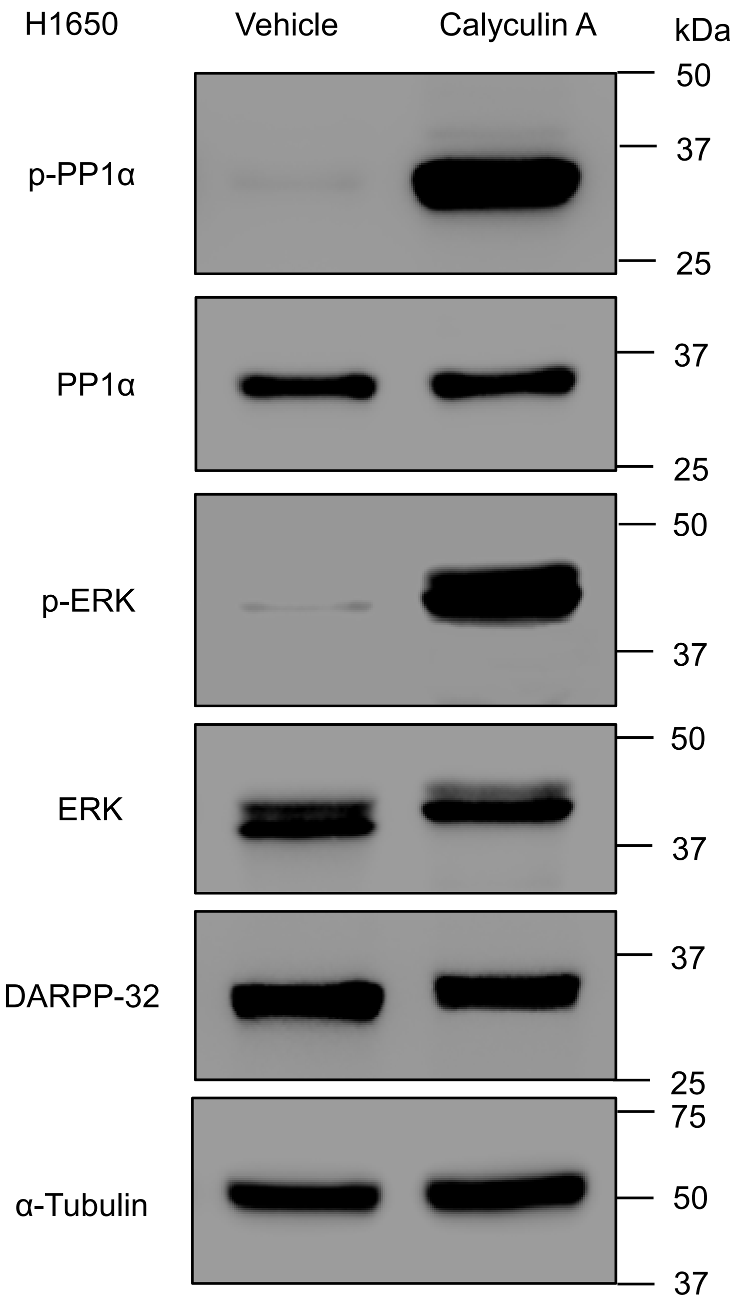

Supplementary Figure 12: Full-sized scans of immunoblots in Figure 4e

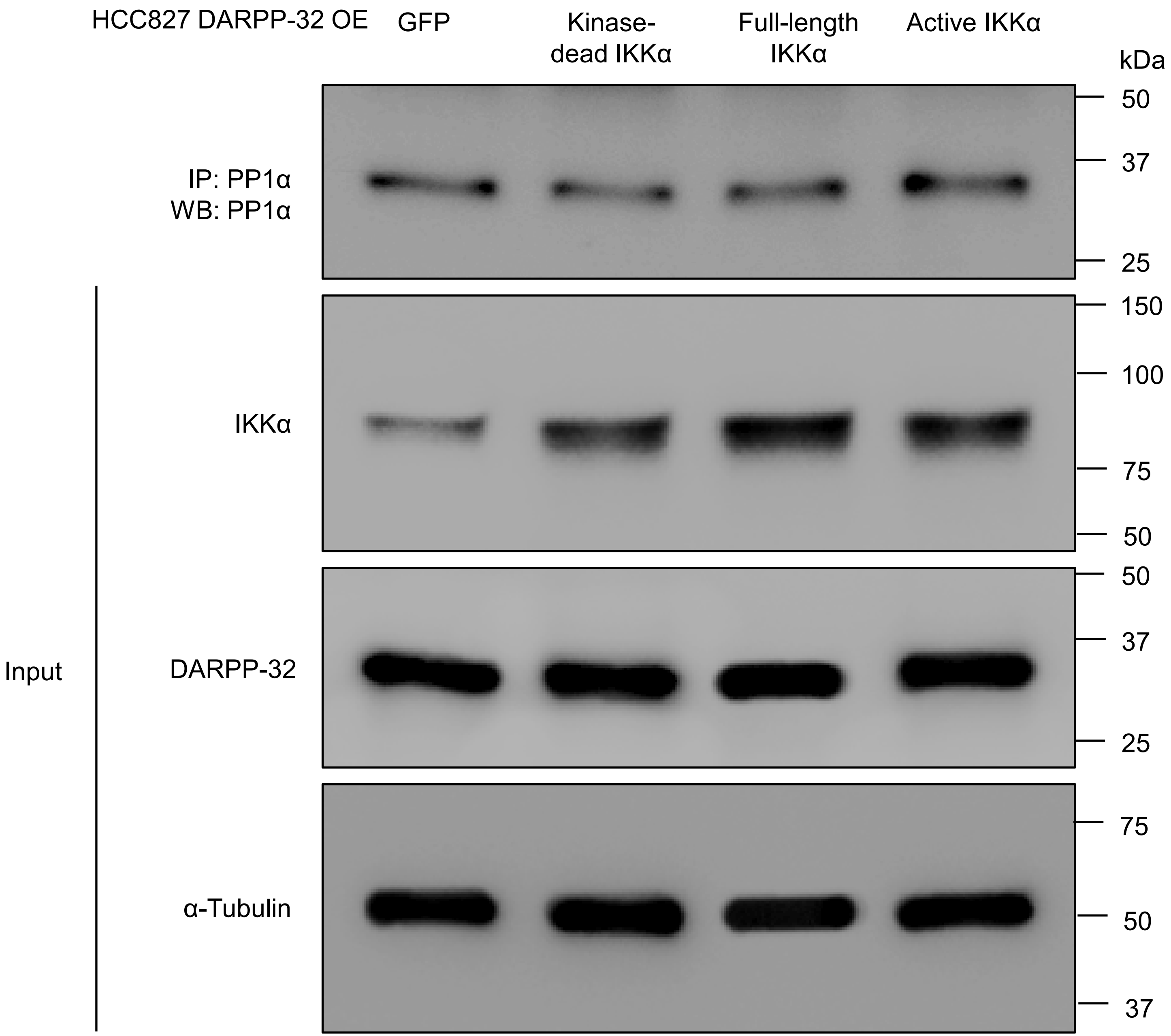

Supplementary Figure 13: Full-sized scans of immunoblots in Figure 4f

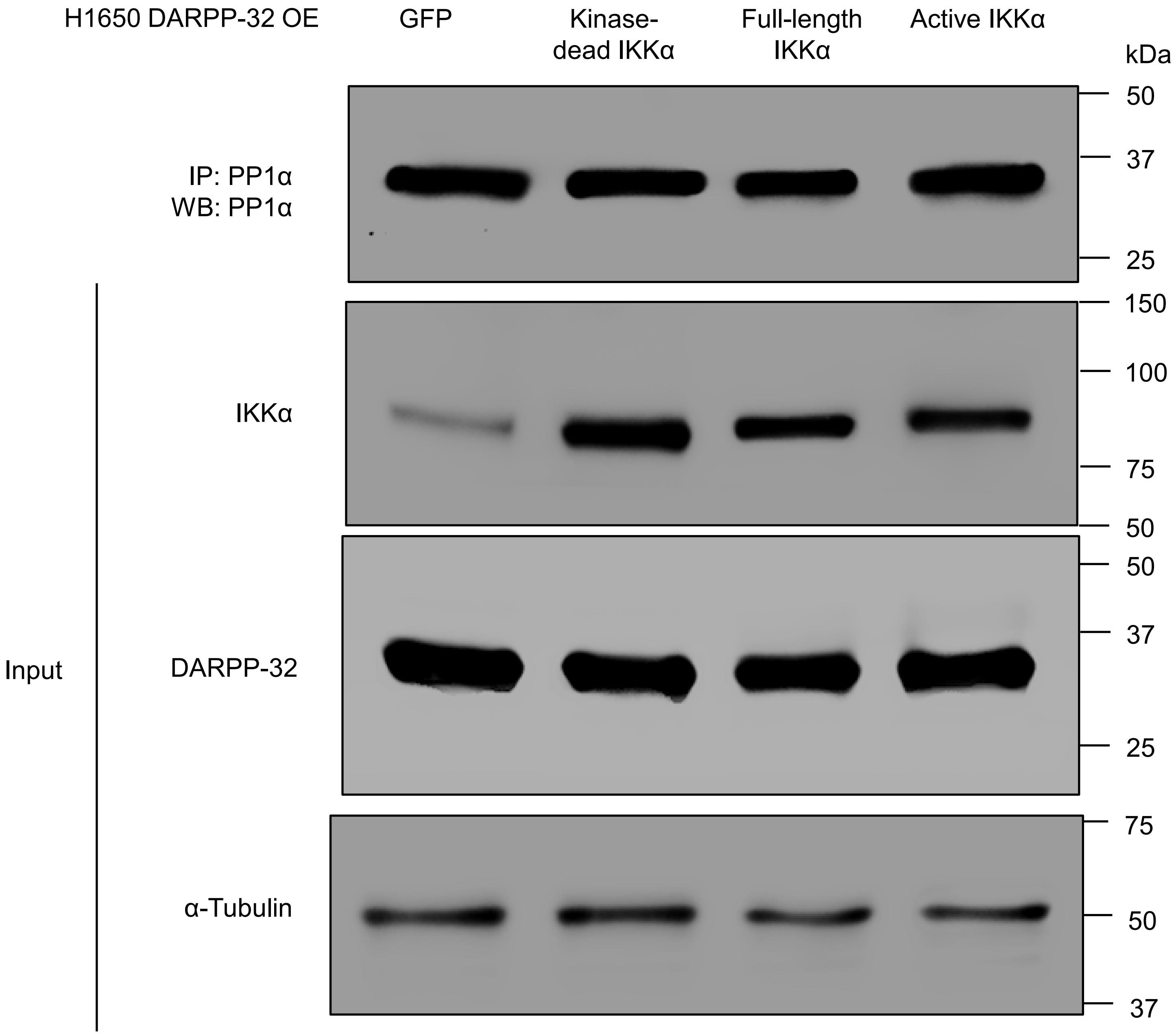

Supplementary Figure 14: Full-sized scans of immunoblots in Figure 4g

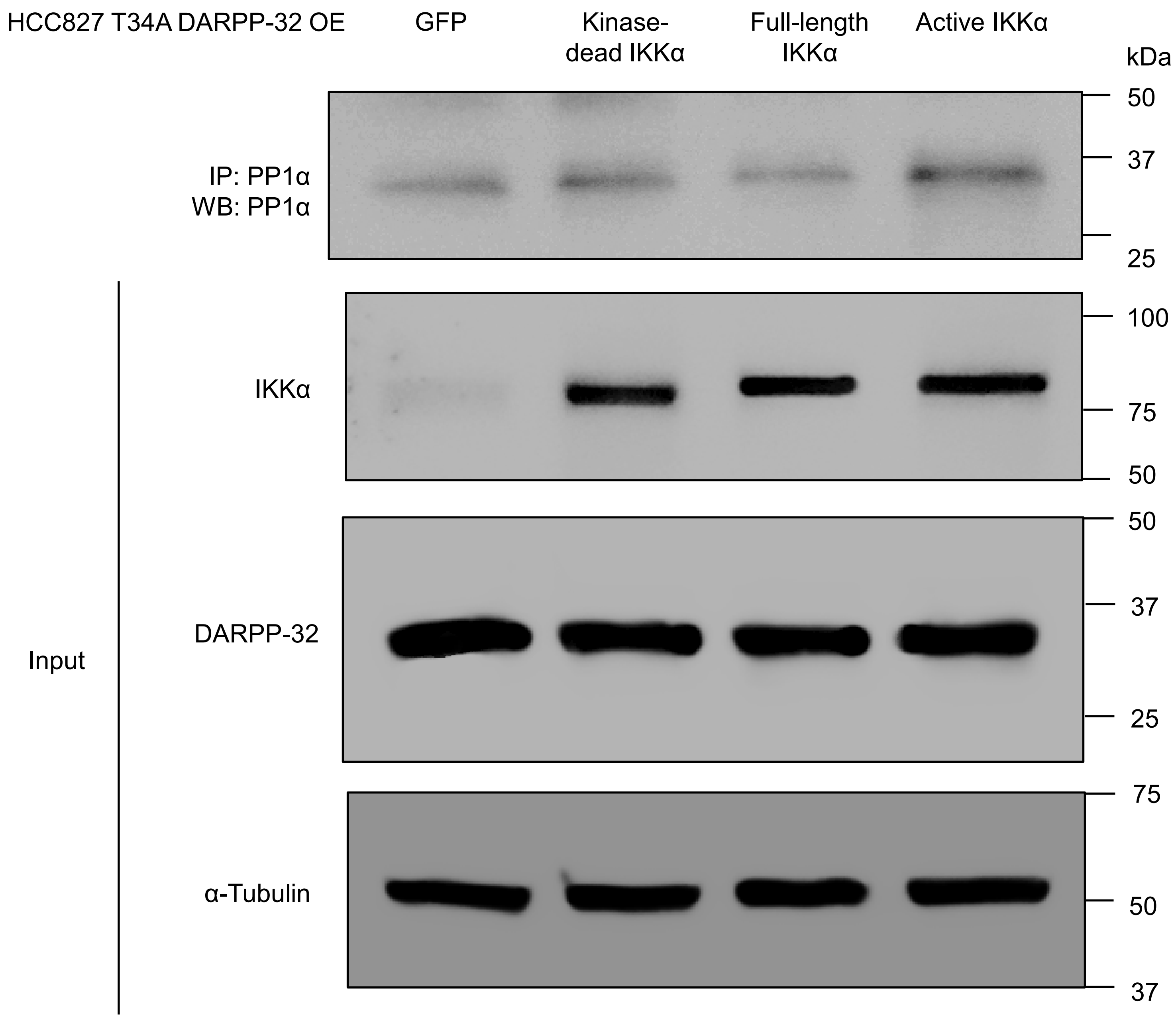

Supplementary Figure 15: Full-sized scans of immunoblots in Figure 4h

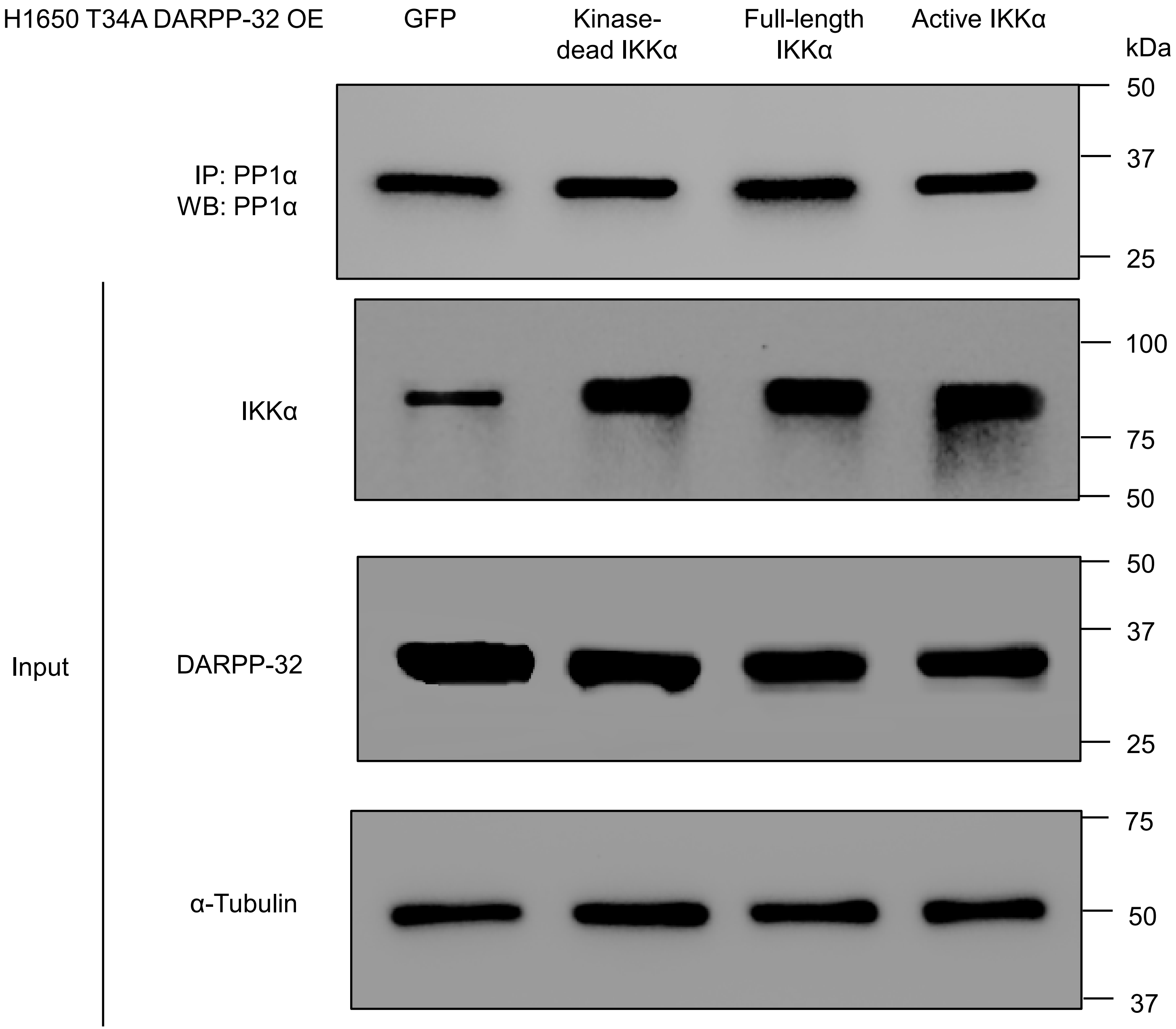

Supplementary Figure 16: Full-sized scans of immunoblots in Figure 5a

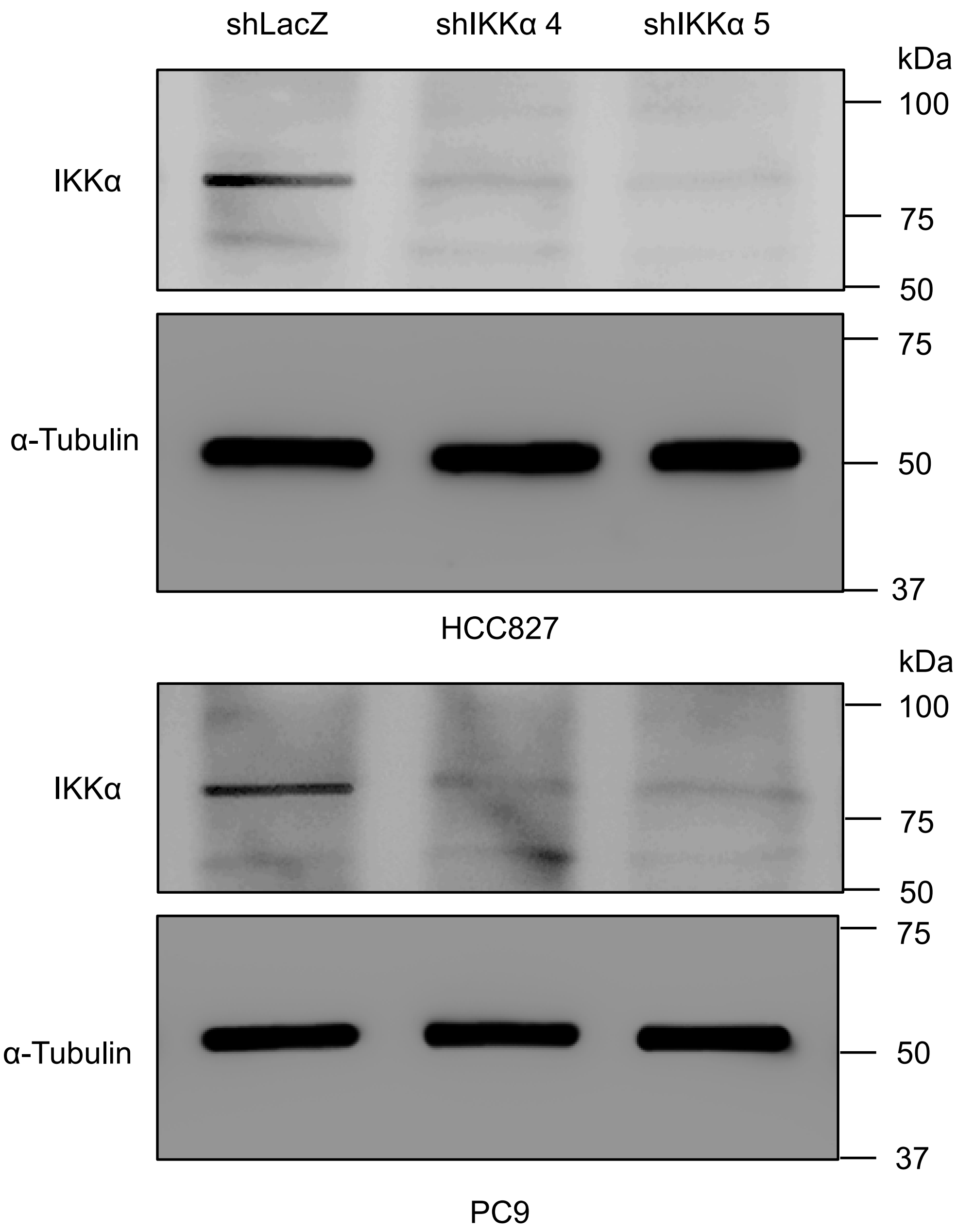

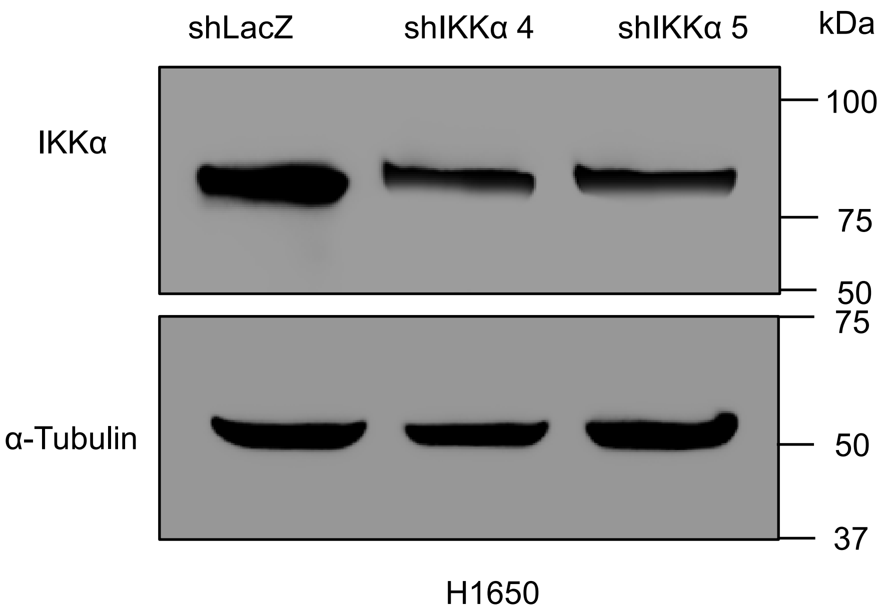

Supplementary Figure 17: Full-sized scans of immunoblots in Figure 5c-d

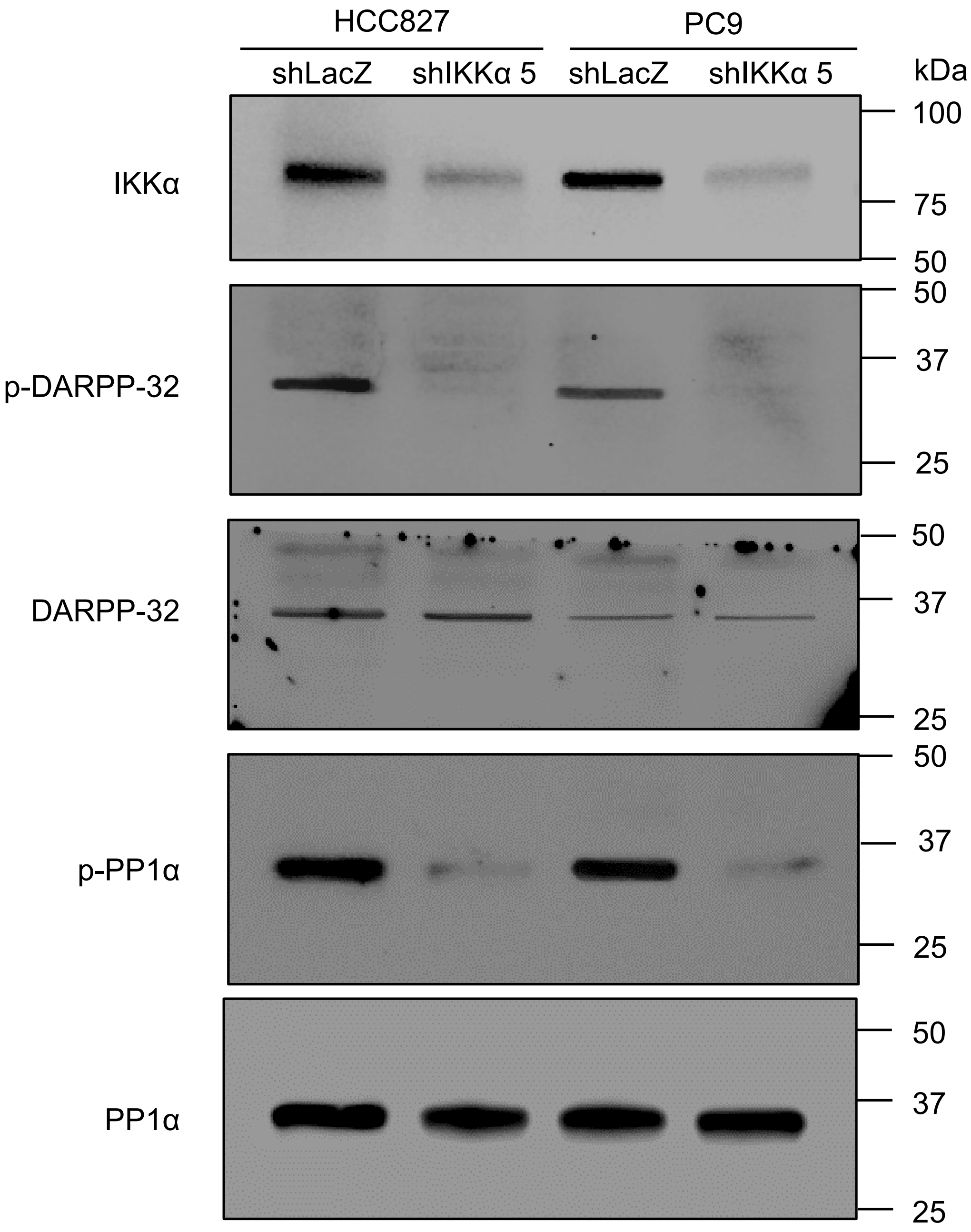

Supplementary Figure 17-continue: Full-sized scans of immunoblots in Figure 5c-d

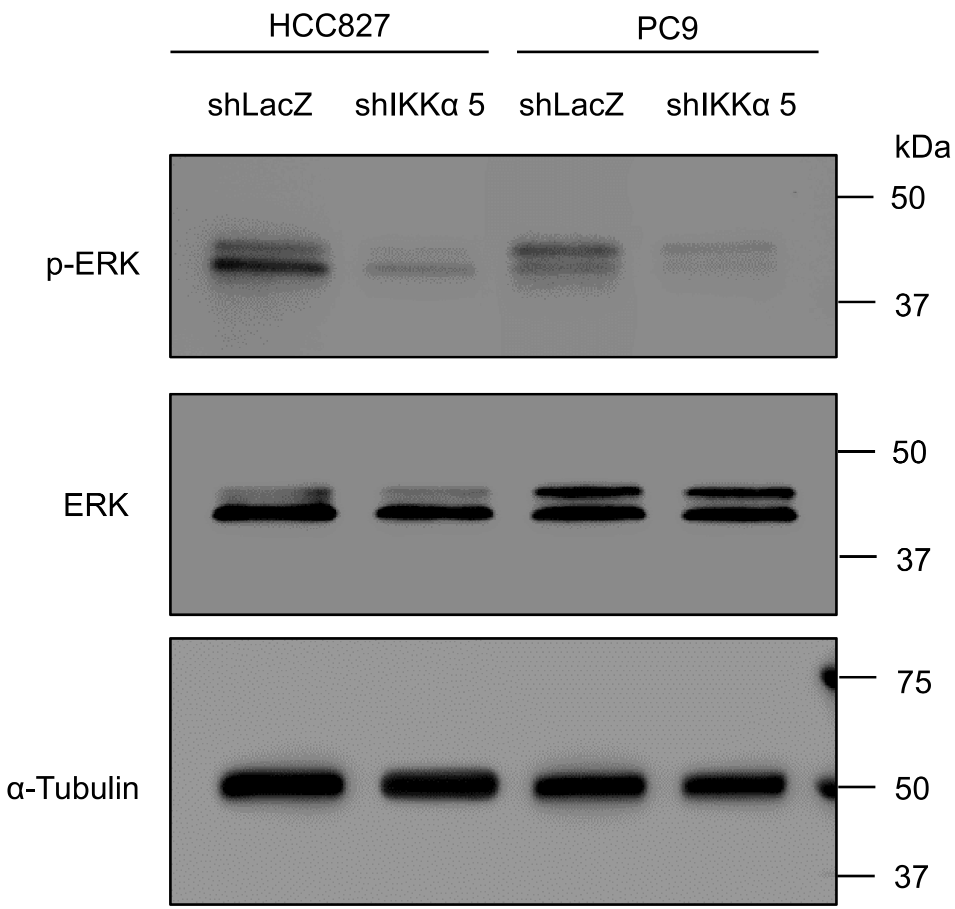

Supplementary Figure 18: Full-sized scans of immunoblots in Figure 6a

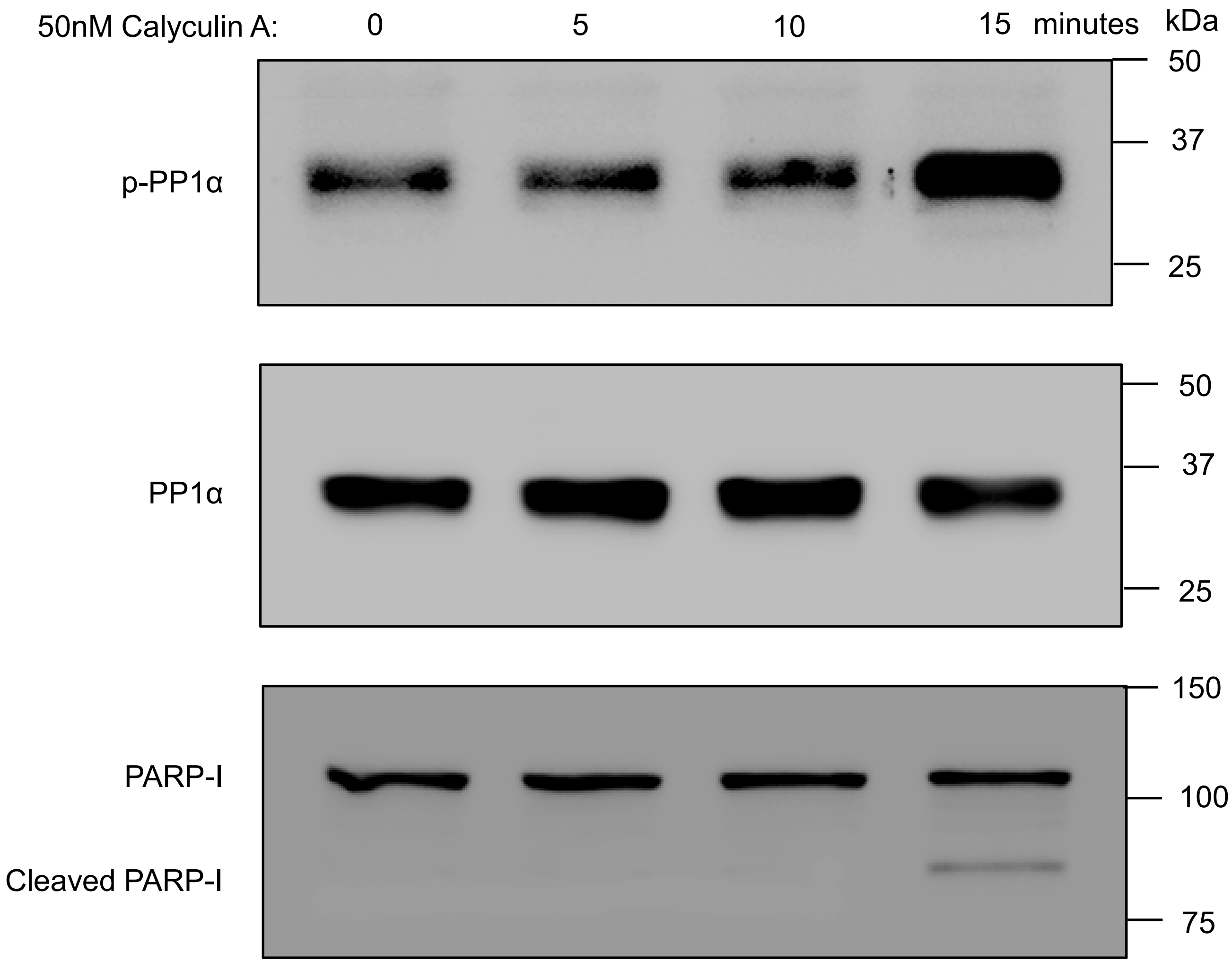

Supplementary Figure 18-continue: Full-sized scans of immunoblots in Figure 6a

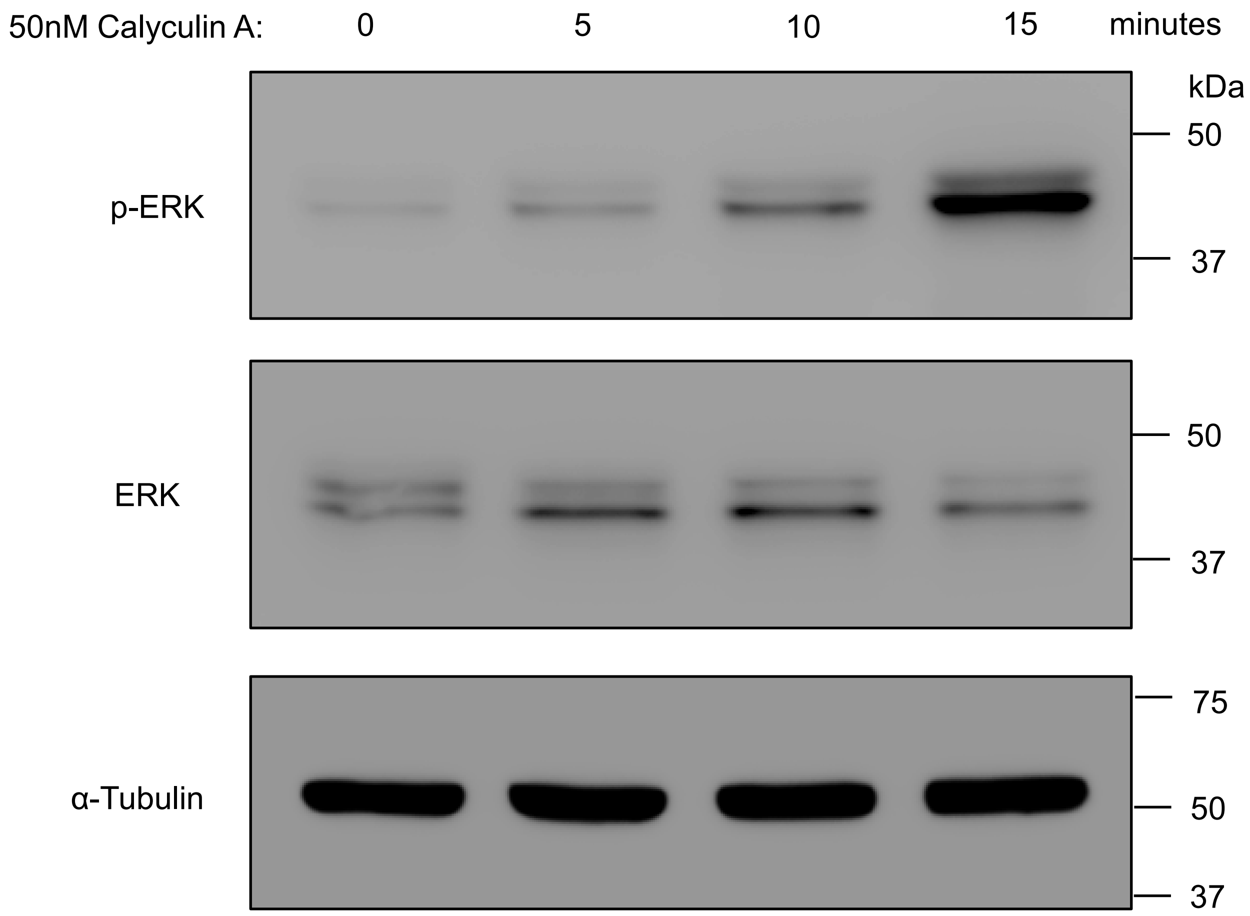

Supplementary Figure 19: Full-sized scans of immunoblots in Figure 7c

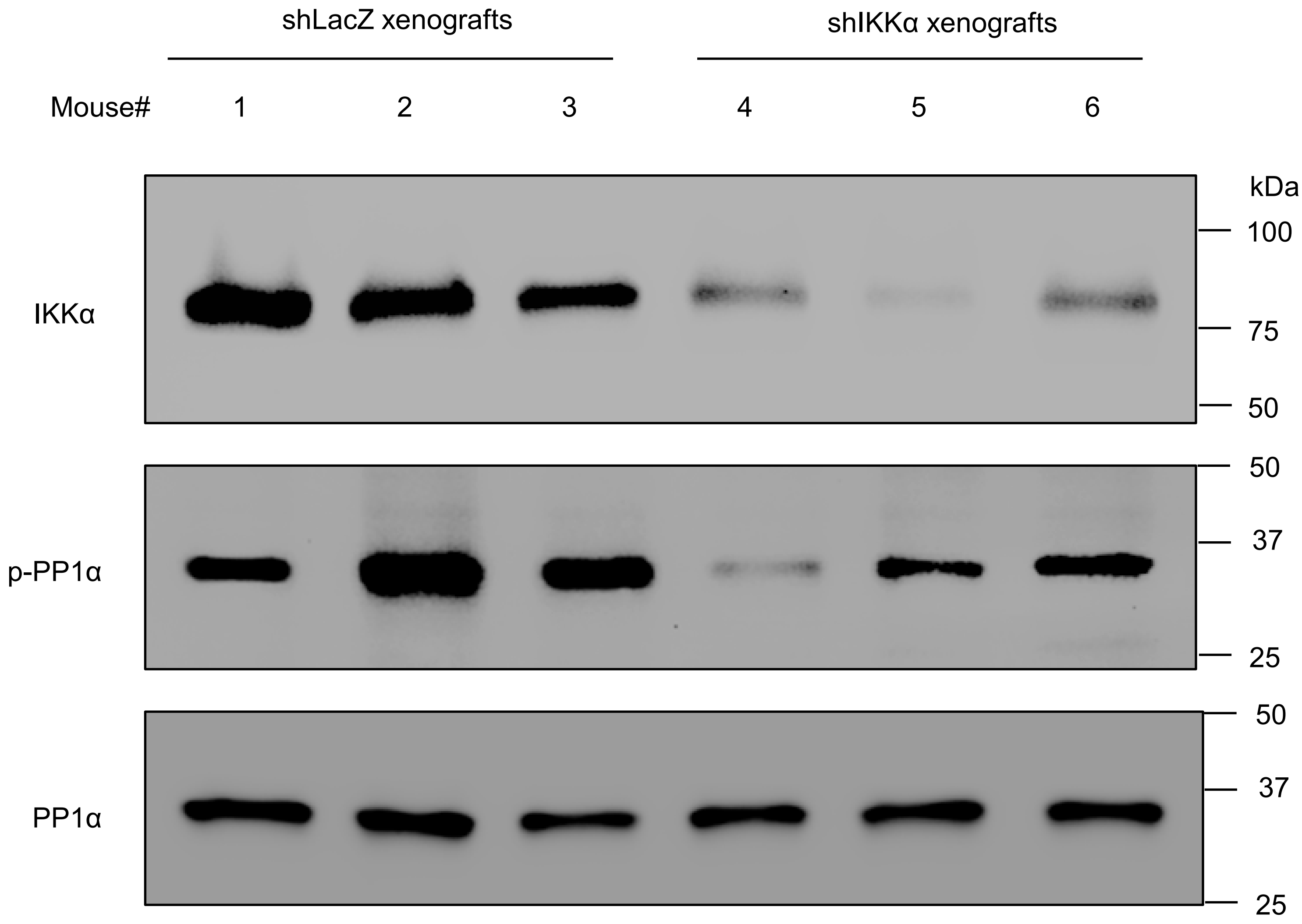

Supplementary Figure 19-continue: Full-sized scans of immunoblots in Figure 7c

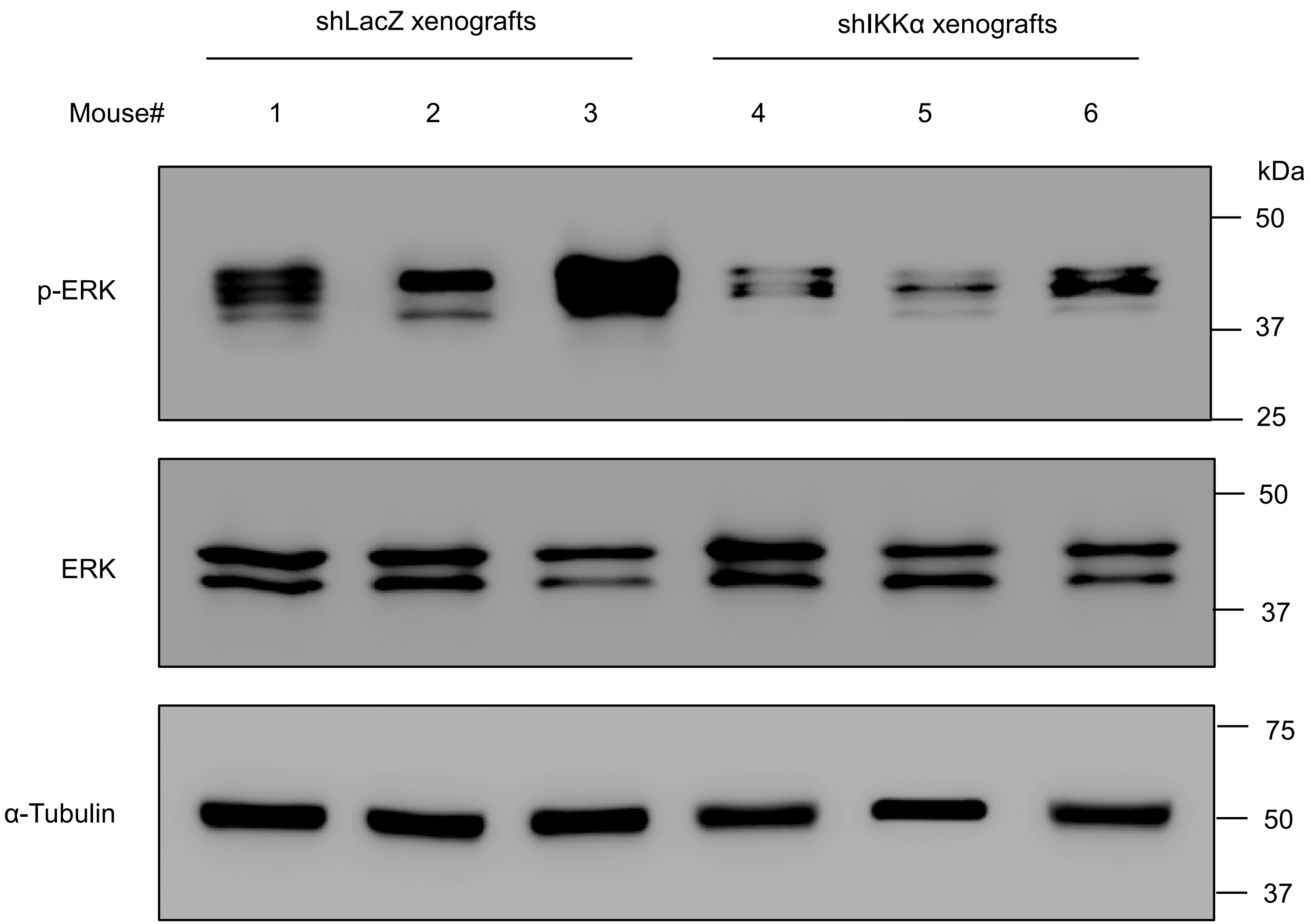

Supplementary Figure 20: Full-sized scans of immunoblots in Supplementary Figure 1a

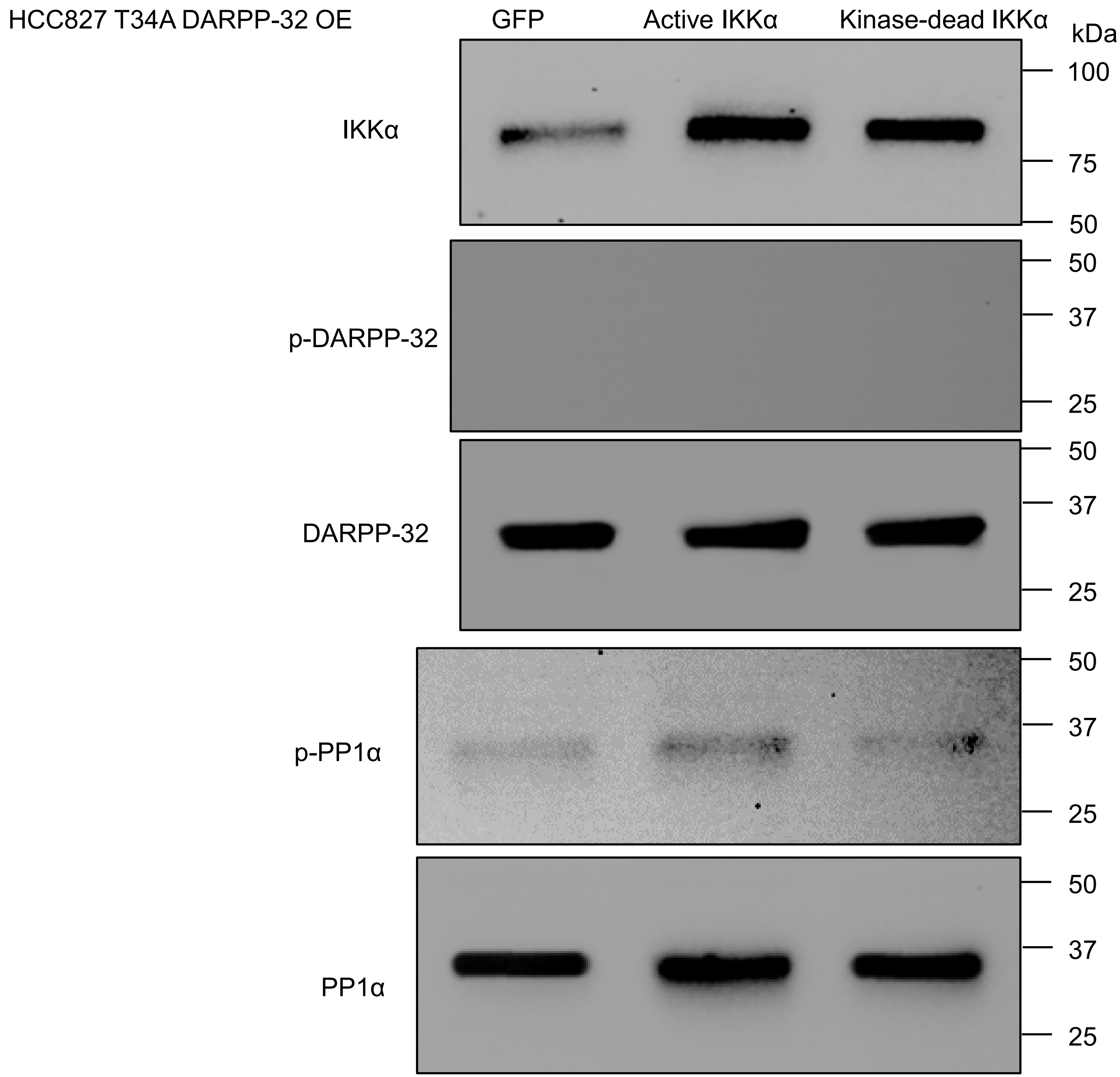

Supplementary Figure 20-continue: Full-sized scans of immunoblots in Supplementary Figure 1a

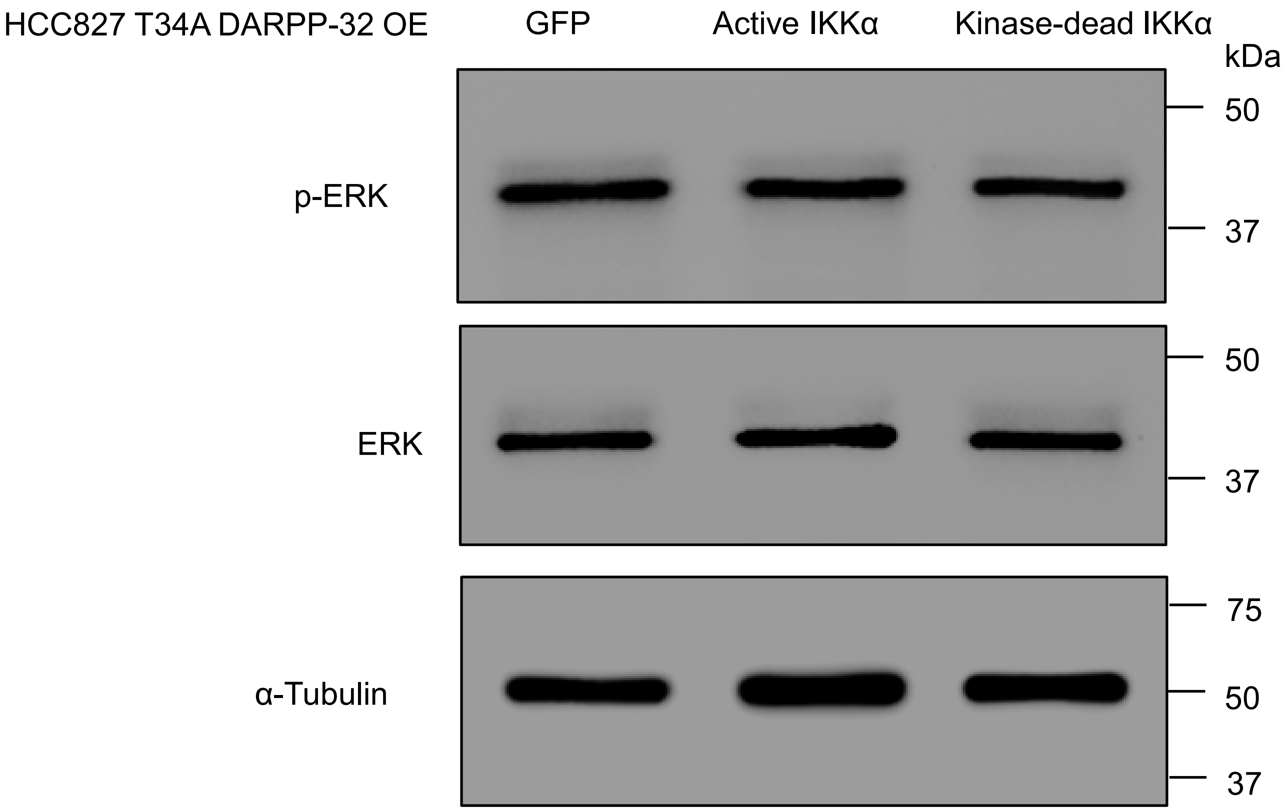

Supplementary Figure 21: Full-sized scans of immunoblots in Supplementary Figure 1b

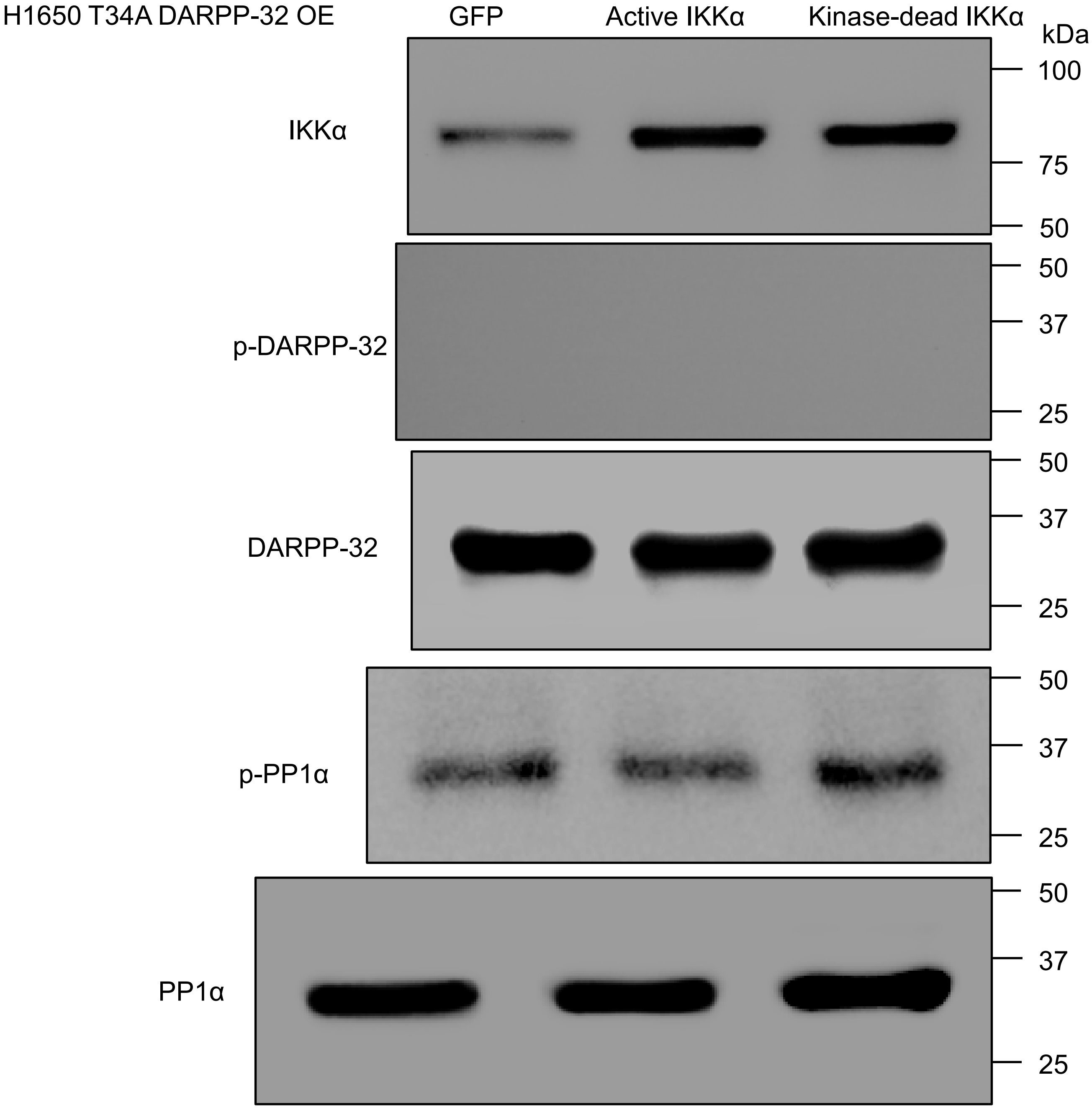

Supplementary Figure 21-continue: Full-sized scans of immunoblots in Supplementary Figure 1b

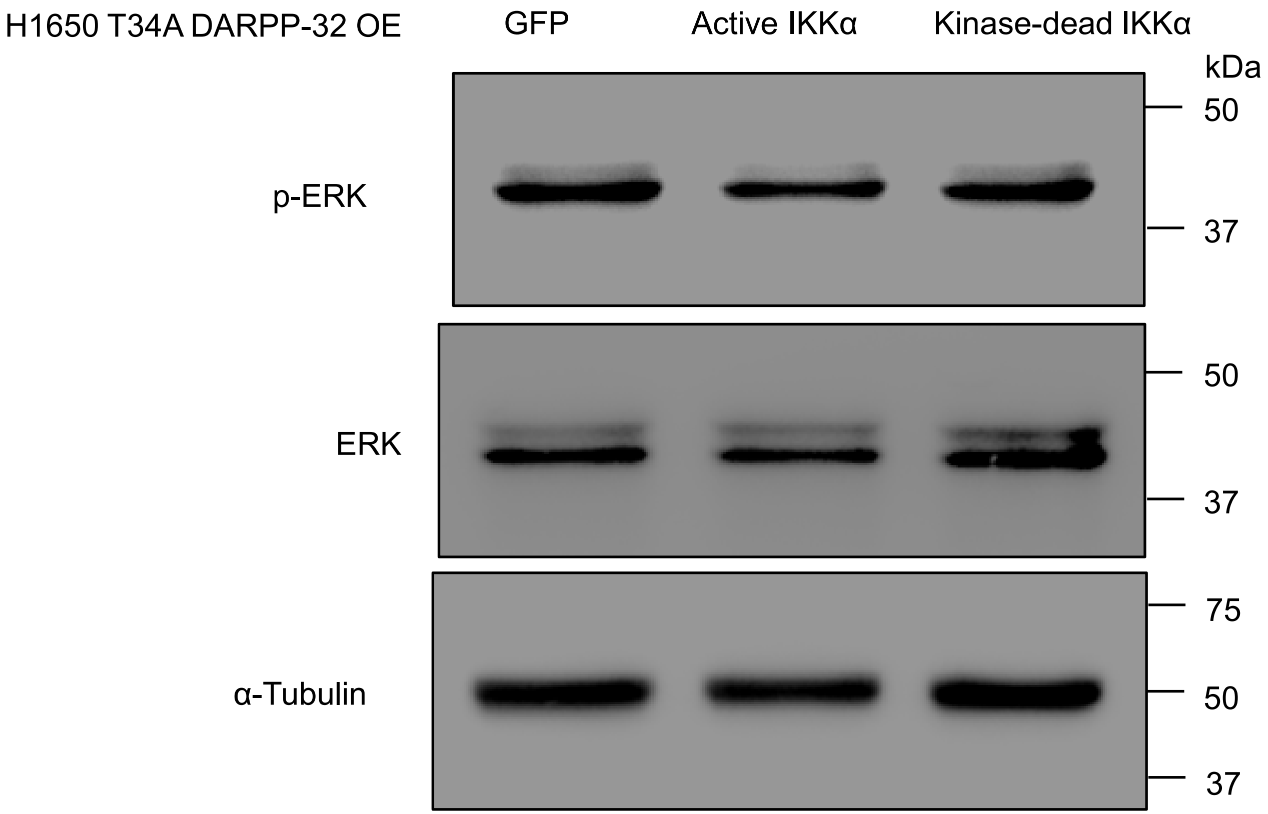

Supplement: Supplementary file 2 — Supplementary Figures [file 41698_2023_370_MOESM2_ESM.pdf]
